# Anti-nephrin antibodies are not enriched in patients with primary and posttransplant recurrent podocytopathies

**DOI:** 10.1172/JCI204727

**Published:** 2026-04-28

**Authors:** Francesco Pecoraro, Luca Perico, Federica Casiraghi, Paola Rizzo, Matias Trillini, Andrea Angeletti, Manuel Alfredo Podestà, Xhuliana Kajana, Agnese Spennacchio, Marta Todeschini, Marilena Mister, Giuseppe Castellano, Ariela Benigni, Giuseppe Remuzzi

**Affiliations:** 1 Department of Molecular Medicine and; 2 Clinical Research Center for Rare Diseases “Aldo e Cele Daccò,” Istituto di Ricerche Farmacologiche Mario Negri IRCCS, Bergamo, Italy.; 3 Division of Nephrology, Dialysis, and Transplantation, IRCCS Istituto Giannina Gaslini, Genoa, Italy.; 4 Renal Research Laboratory, Unit of Nephrology, Dialysis, and Renal Transplantation, Fondazione IRCCS Ca’ Granda Ospedale Maggiore Policlinico, Milan, Italy.; 5 Department of Clinical Sciences and Community Health, University of Milan, Milan, Italy.

**Keywords:** Autoimmunity, Nephrology, Immunoglobulins

## Abstract

**BACKGROUND:**

Anti-nephrin autoantibodies have emerged as a putative pathogenic driver in a subset of patients with podocytopathies, including those with posttransplant disease recurrence.

**METHODS:**

We measured anti-nephrin autoantibodies in a cohort of 65 patients with podocytopathy associated with steroid-sensitive nephrotic syndrome (*n =* 39) and steroid-resistant nephrotic syndrome (*n =* 26) and in 34 patients with posttransplant podocytopathy recurrence. Fourteen patients with membranous nephropathy and 20 healthy volunteers served as controls. ELISA and immunoprecipitation assays were performed to detect anti-nephrin IgG using 2 different recombinant human nephrin proteins. Immunofluorescence analysis was performed to assess gG deposition and its colocalization with nephrin in renal biopsies.

**RESULTS:**

When using an ELISA based on murine cell-derived human antigen, the highest positivity was found in healthy volunteers (55%), correlating with levels of circulating natural anti–α-galactose-α-1,3-galactose antibodies. This cross-reactivity was abrogated with recombinant human nephrin expressed in human cells. In this setting, very low prevalence (<5%) of anti-nephrin antibody-positive patients was found in steroid-sensitive and -resistant nephrotic syndrome cohorts and in patients with posttransplant disease recurrence. These frequencies were comparable to healthy volunteers. Using confocal and super-resolution microscopy, only trace amounts of IgM, but no IgG, were found in the glomeruli of analyzed biopsies, which did not colocalize with nephrin.

**CONCLUSION:**

With the methodology presented here, anti-nephrin reactivity was extremely rare and occurred at comparably low frequencies in healthy controls, native-kidney podocytopathies, and posttransplant disease recurrence. This suggests that these autoantibodies are not inherently disease specific and may not serve as a broad biomarker across podocytopathies.

**TRIAL REGISTRATION:**

ClinicalTrials.gov NCT06334692.

**FUNDING:**

The Medici di Marignano family.

## Graphical abstract

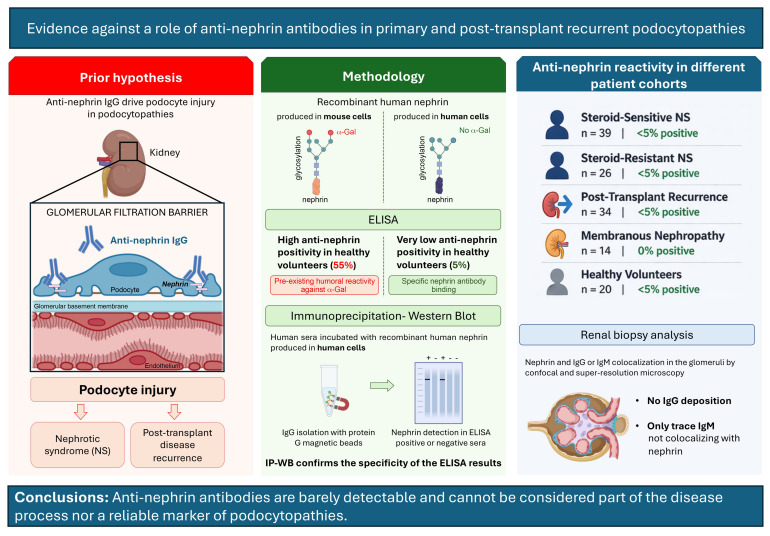

## Introduction

Podocytopathies are a group of glomerular diseases characterized by primary damage to podocytes (1), highly specialized cells that line the outer aspect of the glomerular basement membrane and play a crucial role in renal filtration (2). The most common histological findings are minimal change disease and focal segmental glomerulosclerosis, both associated with nephrotic syndrome (1, 3, 4).

Primary podocytopathies associated with focal segmental glomerulosclerosis are characterized by podocyte injury and massive proteinuria, often culminating in end-stage renal disease. These cases are particularly challenging, as they recur in 30%–50% of kidney transplant recipients (5, 6). This is a major cause of graft dysfunction and failure, typically presenting as severe nephrotic-range proteinuria shortly after transplantation. Podocytopathy recurrence following transplant points to the possibility that circulating factors play a role in disease pathogenesis, which remains incompletely understood (7).

Since an initial report was published in 2022 (8), a series of studies have suggested that anti-nephrin autoantibodies may be a key circulating factor that contributes to podocytopathies (9–11). Nephrin is a critical transmembrane protein in the slit diaphragm of podocytes that preserves the structural and functional integrity of the filtration barrier (2). It has been suggested that anti-nephrin antibodies disrupt this integrity through the induction of nephrin phosphorylation, leading to podocyte detachment, cytoskeletal reorganization, foot process effacement, and increased glomerular permeability, ultimately resulting in proteinuria (9). Across different studies, circulating anti-nephrin autoantibodies have been detected with a highly variable incidence, between 29% and 90% in patients with a minimal change disease pattern and between 9% and nearly 70% in those with renal biopsy findings, consistent with focal segmental glomerulosclerosis (8–12). The presence of circulating anti-nephrin antibodies has been associated with punctate IgG staining colocalizing with nephrin in kidney biopsies. Anti-nephrin antibodies appeared to be markers of disease activity (9) and predictors of posttreatment outcomes (13, 14).

Building on this evidence, recent clinical studies also demonstrated the relevance of circulating anti-nephrin antibodies in patients with podocytopathy recurrence following renal transplant (15, 16). Shirai and colleagues found that 100% of pediatric patients with posttransplant disease recurrence had anti-nephrin antibodies before transplantation or during recurrence, while none of the nonrecurrent patients or controls tested positive (15). In a subsequent study, Batal et al. found lower positivity (38%) for anti-nephrin antibodies in pretransplant samples from patients who experienced posttransplant recurrence (16). Notably, none of the patients without recurrence tested positive for anti-nephrin antibodies (16). Patients with positive pretransplant anti-nephrin antibodies exhibited a trend toward earlier recurrence compared with patients with negative antibodies (16). Biopsies positive for anti-nephrin IgG exhibited nephrin tyrosine phosphorylation, altered distribution, and intense foot process effacement (15). Anti-nephrin autoantibody levels often decrease in remission following therapeutic interventions like plasmapheresis, rituximab, or intensified immunosuppression, suggesting they could be biomarkers for disease monitoring (15).

In the present study, we aimed to investigate the presence of anti-nephrin antibodies in patients with primary podocytopathies in native kidneys as well as in patients with or without posttransplant podocytopathy recurrence. Additionally, individuals with an unrelated glomerular condition, membranous nephropathy (MN), and healthy volunteers (HVs) were included as controls. To this end, we employed a range of complementary techniques, including ELISA with nephrin from different sources as coating antigen, immunoprecipitation, Western blotting, and immunofluorescence to assess the reliability of anti-nephrin antibody detection in these patients.

## Results

### Patient selection and general clinical characteristics.

**Figure 1:**
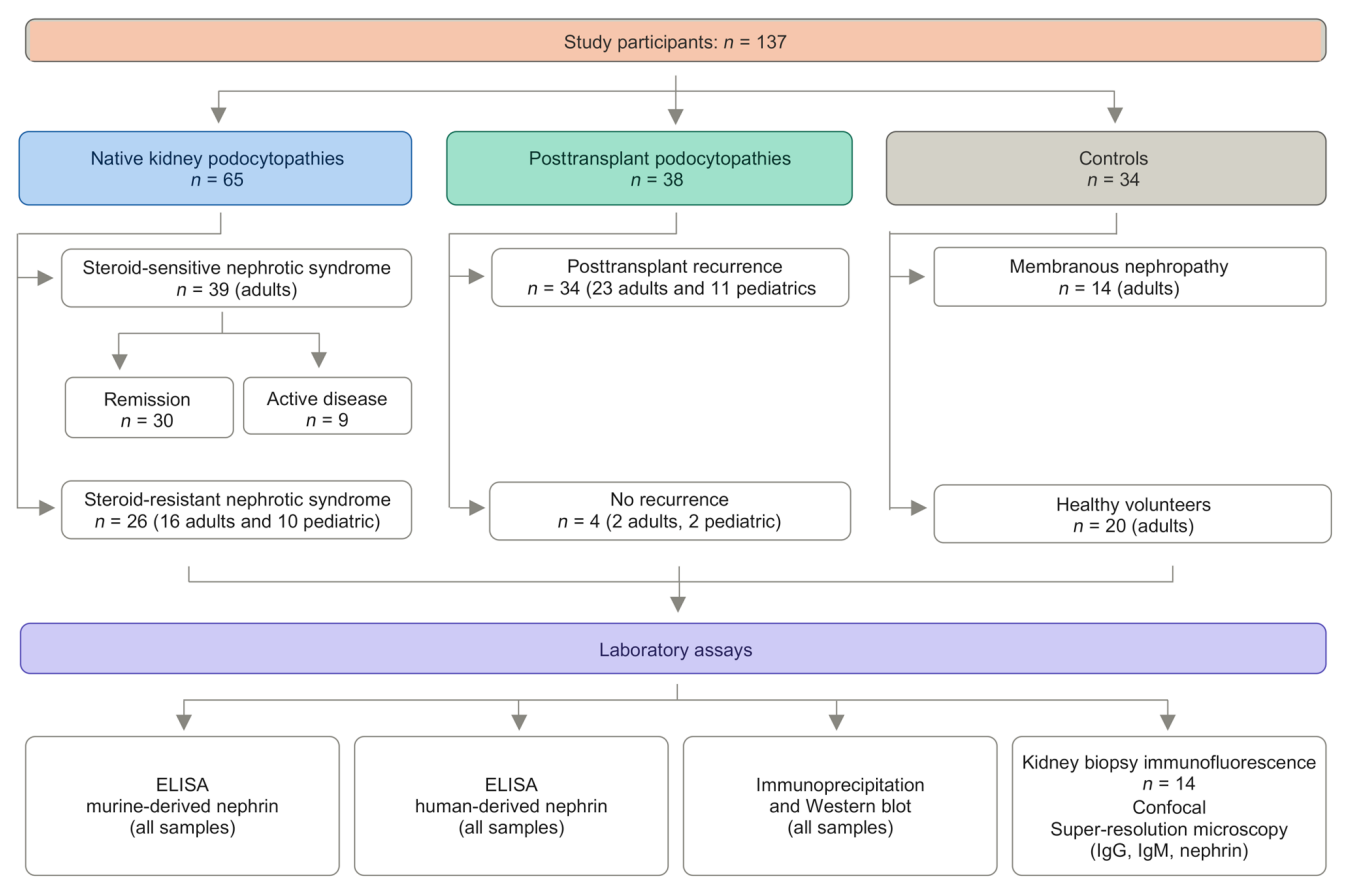
Study flow diagram. A total of 137 participants were included across 2 podocytopathies patient groups (native kidney and posttransplant), a disease control group (MN), and HVs. Native kidney podocytopathies comprised 39 adults with steroid-sensitive nephrotic syndrome (30 sampled during remission and 9 during active disease) and 26 patients with steroid-resistant nephrotic syndrome (16 adults and 10 pediatric). Posttransplant podocytopathies comprised 34 patients with disease recurrence and 4 patients without recurrence after transplantation. All serum samples were tested by indirect ELISA using recombinant human nephrin produced in murine cells and in human embryonic kidney cells. To confirm ELISA results, all serum samples were tested by IP-WB. Available frozen renal biopsies from a subset of 14 podocytopathy patients (*n =* 5 with steroid-sensitive nephrotic syndrome, *n =* 3 with steroid-resistant nephrotic syndrome, and *n =* 6 with posttransplant podocytopathy recurrence) were analyzed by confocal immunofluorescence and super-resolution SIM for IgG, IgM, and nephrin localization. A biopsy from 1 patient with MN was used as a positive technical control for the tissue-based immunofluorescence workflow, with IgG localization assessed together with phospholipase A2 receptor.

As shown in Figure 1, we included 39 adult patients with native steroid-sensitive nephrotic syndrome (Table 1), as defined in KDIGO guidelines (17). For this patient group, serum samples were collected during steroid-induced remission (*n =* 30) or during active disease (*n =* 9). We also included 26 patients with native primary podocytopathies and steroid-resistant nephrotic syndrome (*n =* 16 adult and *n =* 10 pediatric patients), who were negative for monogenic variants associated with nephrotic syndrome (Figure 1 and Table 1).

Additionally, 34 patients with posttransplant podocytopathy recurrence were included in this study (*n =* 23 adult and *n =* 11 pediatric patients; Figure 1 and Table 2). Of these patients, 5 were analyzed prior to transplantation; 7 (*n =* 4 adult and *n =* 3 pediatric patients) were sampled during disease recurrence, within 10 days of diagnosis, i.e., the window in which anti-nephrin antibodies would be expected to peak if they are pathogenic; and 22 were evaluated posttransplant, including 5 on dialysis and 17 (*n =* 11 adult and *n =* 6 pediatric patients) with a functioning graft (serum creatinine levels ranging from 0.5 to 4.75 mg/dL). Details on immunosuppressive therapy at the time of blood collection for patients analyzed during recurrence and for nondialysis patients sampled posttransplant are reported in Supplemental Table 1 (supplemental material available online with this article; https://doi.org/10.1172/JCI204727DS1). Blood collection from patients analyzed during recurrence was performed before interventions such as plasmapheresis or rituximab administration. Lastly, 4 patients (*n =* 2 adult and *n =* 2 pediatric patients) with primary podocytopathies who did not experience recurrence after kidney transplantation were also included (Table 1).

Serum samples from 14 patients with biopsy-proven, active MN (*n =* 12 patients positive for anti–phospholipase A2 receptor antibody and *n =* 2 negative) and 20 HVs (Figure 1 and Table 1) served as controls.

### Anti-nephrin autoantibodies detected by ELISA using nephrin derived from a mouse cell line reflected cross-reactivity with natural α-galactose-α-1,3-galactose antibodies.

**Figure 2:**
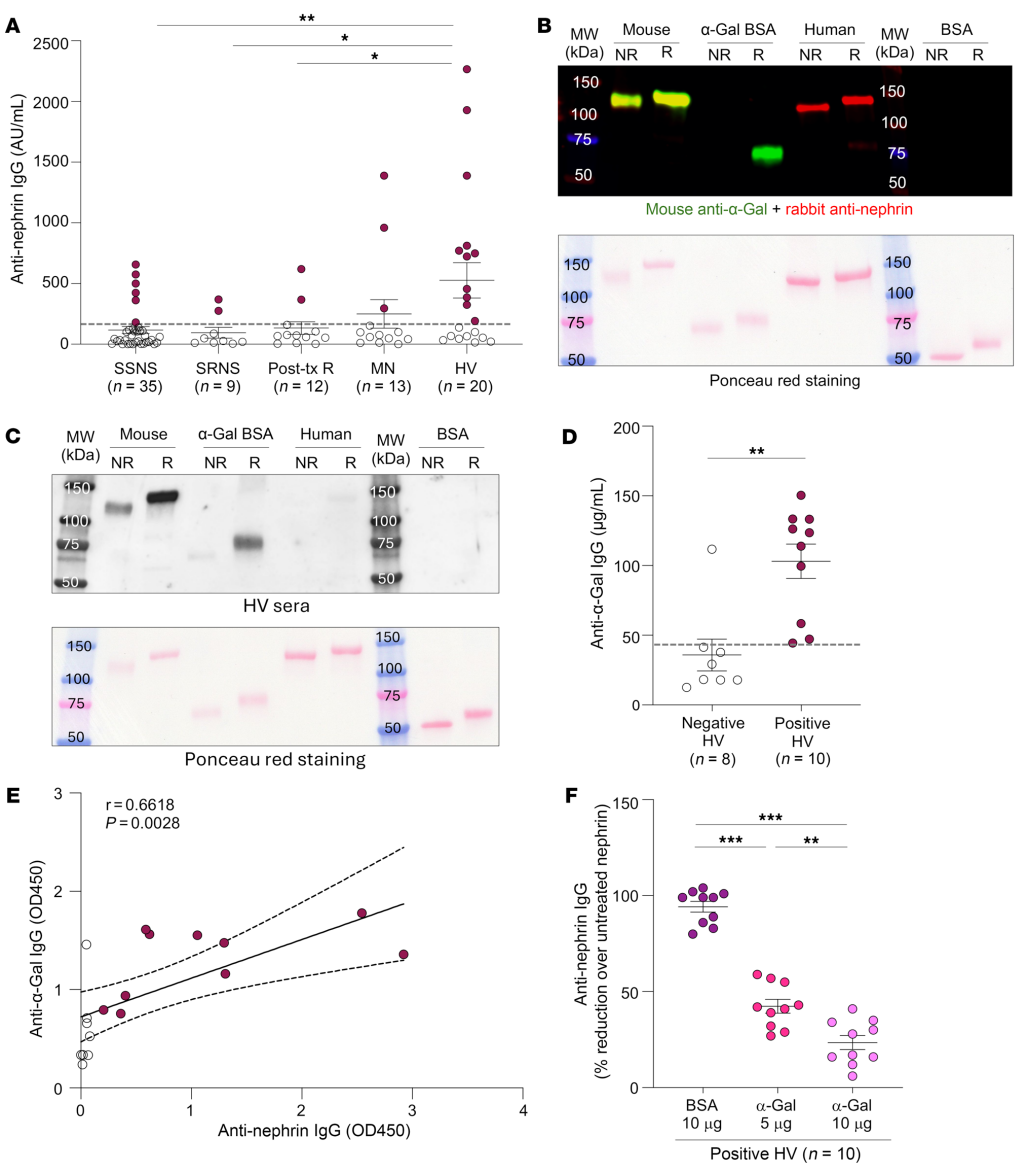
Anti-nephrin antibody detection by ELISA and anti–α-Gal antibody cross-reactivity using human nephrin produced in murine cells. (**A**) Antibodies against the extracellular domain of recombinant nephrin produced in mouse cells were measured by ELISA in patients with steroid-sensitive nephrotic syndrome (SSNS), steroid-resistant nephrotic syndrome (SRNS), and posttransplant podocytopathy recurrence (Post-tx R). HVs and patients with MN served as controls. The dashed line indicates the threshold (180 AU/mL). Positive samples are highlighted in red. (**B** and **C**) Representative Western blot (upper panels) and Ponceau red staining (lower panels) of nephrin produced in murine cells (mouse), α1-3Galβ1-4Glc-BSA (α-Gal–BSA), nephrin produced in human cells (human), and unconjugated BSA under nonreducing (NR) and reducing (R) conditions. Membranes were probed with mouse anti–α-Gal antibody (green) and rabbit anti-nephrin antibody (red) to assess α-Gal modification (*n =* 3) (**B**) or with HV sera (*n =* 4) (**C**) to evaluate the reactivity of circulating antibodies against the different proteins. For each panel, MWs are expressed in kilodaltons. (**D**) Circulating anti–α-Gal antibodies quantified by ELISA in HV sera classified as positive or negative by ELISA with nephrin produced in mouse cells. The dashed line indicates the threshold (42.9 μg/mL). (**E**) Correlation between anti–α-Gal and anti-nephrin absorbance values at OD_450_ in HV sera by ELISA. Linear regression analysis with 95% CI is shown (*r* = 0.6618 and *P* = 0.0028). (**F**) Sera from HV identified as anti-nephrin positive were preabsorbed with unconjugated 10 μg BSA, 5 μg α-Gal–BSA, or 10 μg α-Gal–BSA prior to ELISA with nephrin produced in mouse cells. Data are presented as mean ± SEM and were analyzed using 1-way ANOVA with Tukey’s multiple-comparison test, unpaired 2-tailed *t* test, or paired *t* test, as appropriate. *P < 0.05, **P < 0.01, and ***P < 0.001.

Circulating anti-nephrin antibodies were quantified by standard ELISA with human nephrin from the mouse myeloma cell line, as previously described (8, 15). In the Bergamo adult cohort, 6 out of 35 (17%) patients with steroid-sensitive nephrotic syndrome (1 before immunosuppressive therapy and 5 during steroid-induced remission) tested positive for anti-nephrin antibodies, as did 2 out of 9 (22%) patients with steroid-resistant nephrotic syndrome (1 on steroids and 1 steroid-free; Figure 2A). Additionally, 2 out of 12 (17%) patients with posttransplant podocytopathy recurrence tested positive. For 1 patient, a subsequent serum sample (10 months later) was available, and the test for anti-nephrin antibodies confirmed the persistence of high levels of autoantibodies.

Among the patients with MN, 3 out of 13 (23%) were positive for anti-nephrin antibodies. However, the highest percentage of positive subjects was observed in the HV group, with 11 subjects out of 20 (55%) testing positive for circulating anti-nephrin antibodies (Figure 2A). Of note, antibody levels in HVs were significantly higher compared with patients with posttransplant podocytopathy recurrence or steroid-sensitive and -resistant nephrotic syndrome (Figure 2A).

To elucidate the unexpectedly high frequency of anti-nephrin antibody positivity among HVs, we considered recent evidence indicating that proteins expressed in non-human cells can express α-galactose-α-1,3-galactose (α-Gal) residues on their glycan structures (18). Since natural anti–α-Gal antibodies are highly prevalent in humans (19), we hypothesized that the apparent anti-nephrin reactivity observed in control sera could result from cross-reactivity with α-Gal epitopes on recombinant nephrin. To test this hypothesis, we first performed Western blot analysis using a commercially available mouse anti–α-Gal antibody, together with a rabbit anti-human nephrin antibody. As shown in Supplemental Figure 1, nephrin produced in murine cells displayed a clear α-Gal signal (green) that overlapped with nephrin staining (red), generating the characteristic yellow band indicative of colocalization (Figure 2B). Importantly, α-Gal reactivity was observed predominantly under reducing conditions (Figure 2B), supporting a nonconformational recognition. The specificity was confirmed by the similar pattern of reactivity of the antibody toward α1-3Galβ1-4Glc-BSA, used as a positive control (Figure 2B). In contrast, neither nephrin produced in human cells nor normal BSA expressed α-Gal, as revealed by the lack of green antibody signal (Supplemental Figure 1 and Figure 2B).

To determine whether control sera contained α-Gal–reactive antibodies capable of binding nephrin produced in murine cells, we performed the same Western blot using sera from HVs. As shown in Figure 2C, human IgG recognized only the recombinant nephrin produced in murine cells (Figure 2C). Also in this setting, the signal was detectable predominantly under reducing conditions (Figure 2C), consistent with binding to linear α-Gal–containing glycans. HV sera also reacted with α1-3Galβ1-4Glc-BSA (Figure 2C), confirming the presence of natural anti–α-Gal antibodies. In contrast, no IgG reactivity was observed against human nephrin produced in human cells or against unconjugated BSA (Figure 2C), indicating that binding was strictly dependent on α-Gal epitopes.

Next, we developed and optimized an ELISA to quantify anti–α-Gal IgG in available serum samples. HVs positive for anti-nephrin IgG exhibited significantly higher anti–α-Gal IgG titers compared with anti-nephrin–negative individuals (Figure 2D). Receiver operating characteristic curve analysis yielded an AUC of 0.95 (95% CI, 0.722–1.00; *P =* 0.00138), indicating excellent discriminative ability of α-Gal antibody titers to distinguish between anti-nephrin–positive and –negative sera (Supplemental Figure 2). The optimal cutoff value for serum IgG against α-Gal, determined using the Youden index, was 42.9 μg/mL, corresponding to a sensitivity of 100% and a specificity of 87.5%. Consistently, correlation analysis confirmed this relationship, revealing a significant positive correlation between anti–α-Gal and anti-nephrin IgG reactivities in HV sera (Figure 2E; Pearson’s *r* = 0.6618, *P =* 0.0028).

To functionally verify that the anti-nephrin reactivity detected by ELISA was predominantly driven by α-Gal recognition, sera from HVs positive for anti-nephrin antibodies were preabsorbed with BSA (10 μg/mL) or α-Gal–BSA (5 or 10 μg/mL) prior to analysis. Preabsorption of sera with unconjugated BSA did not alter sera reactivity against murine cell–produced nephrin, indicating the absence of nonspecific interference (Figure 2F). In contrast, preabsorption with α-Gal–BSA resulted in a significant and dose-dependent reduction in sera reactivity (Figure 2F).

To provide direct mechanistic evidence that α-Gal moieties on murine cell–derived recombinant nephrin account for the apparent anti-nephrin reactivity observed in HV sera, we performed a series of enzymatic deglycosylation experiments targeting terminal glycan residues. As an initial approach, we evaluated the presence and linkage of α-Gal residues on recombinant human nephrin produced in murine cells under standard denaturing conditions (3 hours at 37°C) in the presence or absence of deglycosidases, including PNGase F (removing N-linked glycans), O-glycosidase (removing core O-linked glycans), and α-neuraminidase (removing terminal sialic acids). Under these conditions, only PNGase F treatment resulted in complete loss of α-Gal signal (Supplemental Figure 3A), indicating that α-Gal residues are predominantly carried on N-linked glycans. However, these denaturing conditions markedly altered nephrin integrity, as evidenced by a pronounced molecular weight shift and fragmentation into lower molecular weight species compared with the expected approximately 150 kDa band (Supplemental Figure 3A). We therefore optimized the experimental conditions by performing PNGase F digestion under nondenaturing conditions. Nephrin was incubated with PNGase F either for 1 hour at 37°C (Supplemental Figure 3B) or for 2 hours at room temperature followed by overnight incubation at 4°C (Supplemental Figure 3C), as previously described (20). Under these conditions, the structural integrity of nephrin was preserved, as confirmed by consistent detection of nephrin with an approximately 150 kDa molecular weight (red; Supplemental Figure 3, B and C). Nevertheless, although reduced, residual α-Gal reactivity persisted on nephrin, as indicated by overlapping signal in merged channels (yellow; Supplemental Figure 3, B and C). This suggests that the complex conformational structure of nephrin limits the accessibility of PNGase F to all N-linked glycans under native conditions.

To overcome this limitation, we employed α1-3,4,6-galactosidase (α-galactosidase), which selectively removes terminal α-Gal residues without requiring complete glycan removal. As shown in Supplemental Figure 3D, a 1-hour treatment at 37°C was sufficient to completely abolish α-Gal signal on native murine cell–derived nephrin, indicating efficient and specific removal of α-Gal epitopes.

Having identified α-galactosidase as the most effective approach to eliminate α-Gal residues, we next assessed whether degalactosylation affected serum antibody binding. Western blot analysis revealed that sera from HVs recognized murine cell–derived nephrin predominantly under reducing conditions when the protein was left untreated, while α-galactosidase treatment abolished this reactivity (Supplemental Figure 4A). Finally, we performed an ELISA exposing sera from HVs to untreated mouse cell–derived nephrin or α-galactosidase–treated nephrin. We observed a significant reduction of sera reactivity against α-galactosidase–treated nephrin compared with untreated nephrin (Supplemental Figure 4B).

Collectively, these enzymatic deglycosylation experiments provide independent and convergent evidence that the apparent anti-nephrin positivity detected in healthy control sera against murine cell–derived recombinant nephrin is largely driven by recognition of α-Gal–containing glycan epitopes, rather than bona fide anti-nephrin autoantibodies.

### ELISA with nephrin derived from the human cell line did not confirm detection of anti-nephrin antibodies by ELISA with nephrin derived from a murine cell line.

Having demonstrated that human nephrin produced in murine cells exhibited cross-reactivity with natural anti–α-Gal antibodies, we next analyzed the sera using human nephrin produced in human cells (20), which did not exhibit this confounding reactivity. To this end, all sera from patients with nephrotic syndrome and MN, as well as HVs, were retested by ELISA using human nephrin obtained from human embryonic kidney cells as coating antigen (Supplemental Figure 5). Additionally, serum samples from external centers were included, comprising 2 cases initially identified as anti-nephrin–positive in the Genoa laboratory and subsequently confirmed in our laboratory using the assay described above.

**Figure 3:**
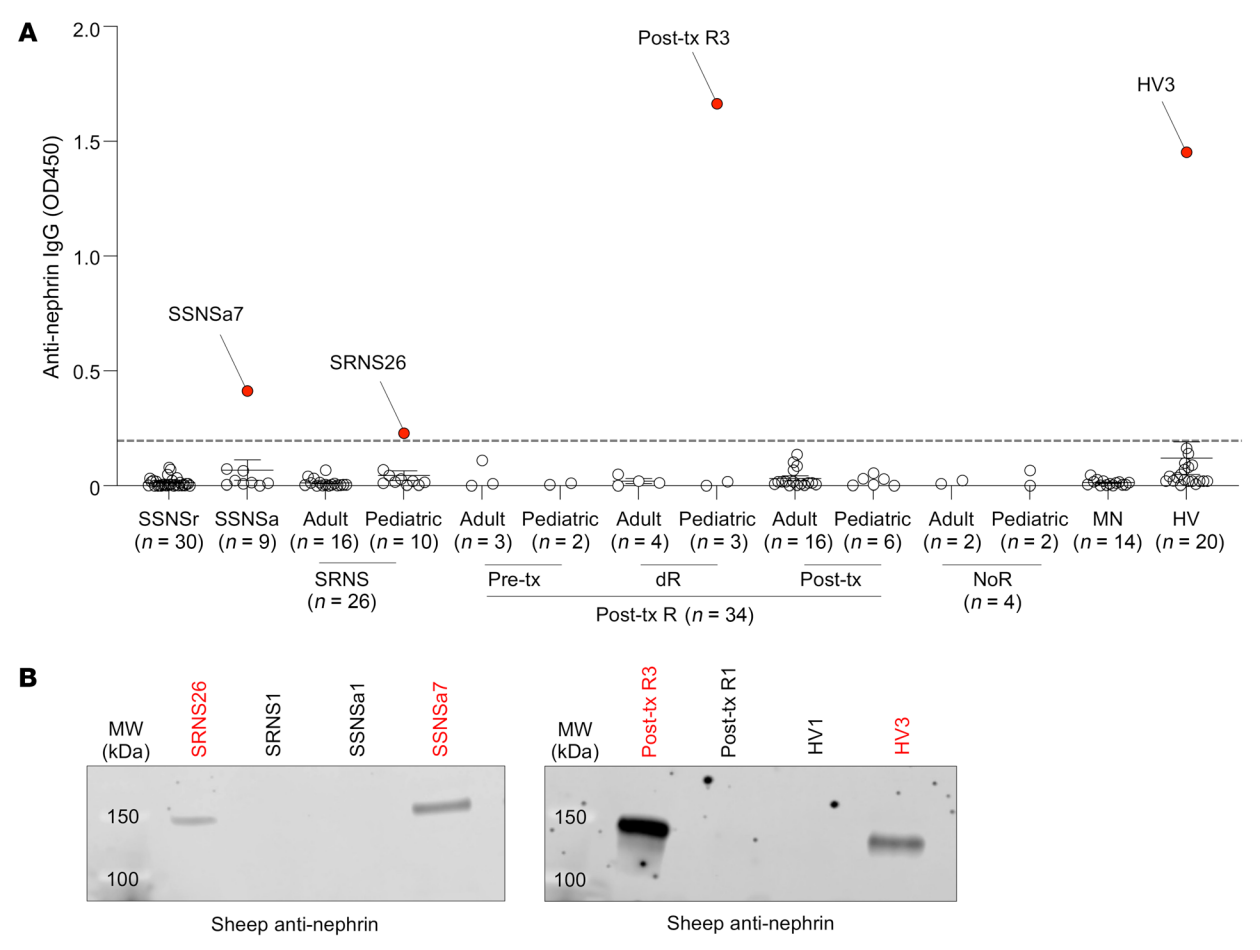
Anti-nephrin antibody detection by ELISA and immunoprecipitation using human nephrin produced in human cells. (**A**) Absorbance values at OD_450_ measured by indirect ELISA using plates coated with the extracellular domain of recombinant human nephrin produced in human cells in patients with SSNS during remission (SSNSr) or active disease (SSNSa), with SRNS, or with (Post-tx R) or without (NoR) posttransplant podocytopathy recurrence. Serum samples from patients with posttransplant podocytopathy recurrence were collected pretransplant (Pre-tx), during disease recurrence (dR), or posttransplant (Post-tx). Patients with MN and HVs served as controls. The dashed line indicates the threshold (0.150 OD). Positive samples are highlighted in red. (**B**) Representative images of IP-WB performed using nephrin produced in human cells. Detection was carried out using a sheep anti-nephrin primary antibody. MWs are expressed in kilodaltons. Shown are the 4 sera that tested positive in **A**. Each positive sample is displayed alongside an ELISA-negative sample from the same corresponding patient category. Complete IP-WB data for the entire cohort are shown in Supplemental Figure 6. Data are presented as mean ± SEM and were analyzed using 1-way ANOVA with Tukey’s multiple-comparison test.

As shown in Figure 3A, among the adult patients with steroid-sensitive nephrotic syndrome, none who were in remission tested positive (SSNSr, 0/30; 0%), whereas 1 of 9 sampled during active disease exhibited low-level reactivity (SSNSa, 1/9; 11%). Similarly, 1 out of 10 pediatric patients with steroid-resistant nephrotic syndrome (SRNS) tested positive for anti-nephrin antibodies, whereas none of the 16 adult SRNS patients were positive, leading to an overall incidence of anti-nephrin antibodies of 4% in SRNS (1/26 patients). Among patients with posttransplant podocytopathy recurrence, only 1 pediatric patient tested positive (1/34; 3%). This patient was sampled 12 days after the diagnosis of disease recurrence, whereas all other patients with recurrent podocytopathy tested negative irrespective of the timing of sampling during the disease course (Figure 3A). No reactivity was detected in nephrotic syndrome patients who did not experience recurrence after transplant (0/4; 0%) nor in patients with MN (0/14; 0%). Finally, 1 of 20 HVs (5%) tested positive with high antibody levels.

To confirm the specificity of the ELISA results, all serum samples were subsequently tested by immunoprecipitation followed by Western blot (IP-WB) using human nephrin produced in human cells. As shown in Figure 3B, every sample identified as positive by ELISA was found positive in IP-WB, and ELISA-negative sera were found negative (Supplemental Figure 6).

To confirm that the reactivity against human cell–derived nephrin reflected true protein-directed antibody binding rather than glycan-dependent cross-reactivity, we performed enzymatic antigen deglycosylation under native conditions (1 hour at 37°C). As expected, nephrin produced in human cells did not display detectable α-Gal residues (Supplemental Figure 7A). A consistent shift in molecular weight of nephrin antigen was observed only following PNGase F treatment, confirming that most of the glycosylation is N-linked (Supplemental Figure 7A). When enzymatically treated nephrin was used as coating antigen in ELISA, the reactivity of 4 positive serum did not change across all conditions (Supplemental Figure 7B).

Collectively, these findings demonstrate that the anti-nephrin reactivity detected using human cell–derived nephrin reflects genuine protein-directed antibody recognition rather than nonspecific glycan-dependent binding. Notably, such reactivity was very rare and occurred at similarly low frequencies across all groups, including healthy individuals, suggesting that these antibodies are not enriched across the spectrum of diseases examined.

### Nephrin is not the primary glomerular target of IgG and IgM in patient renal biopsies.

**Figure 4:**
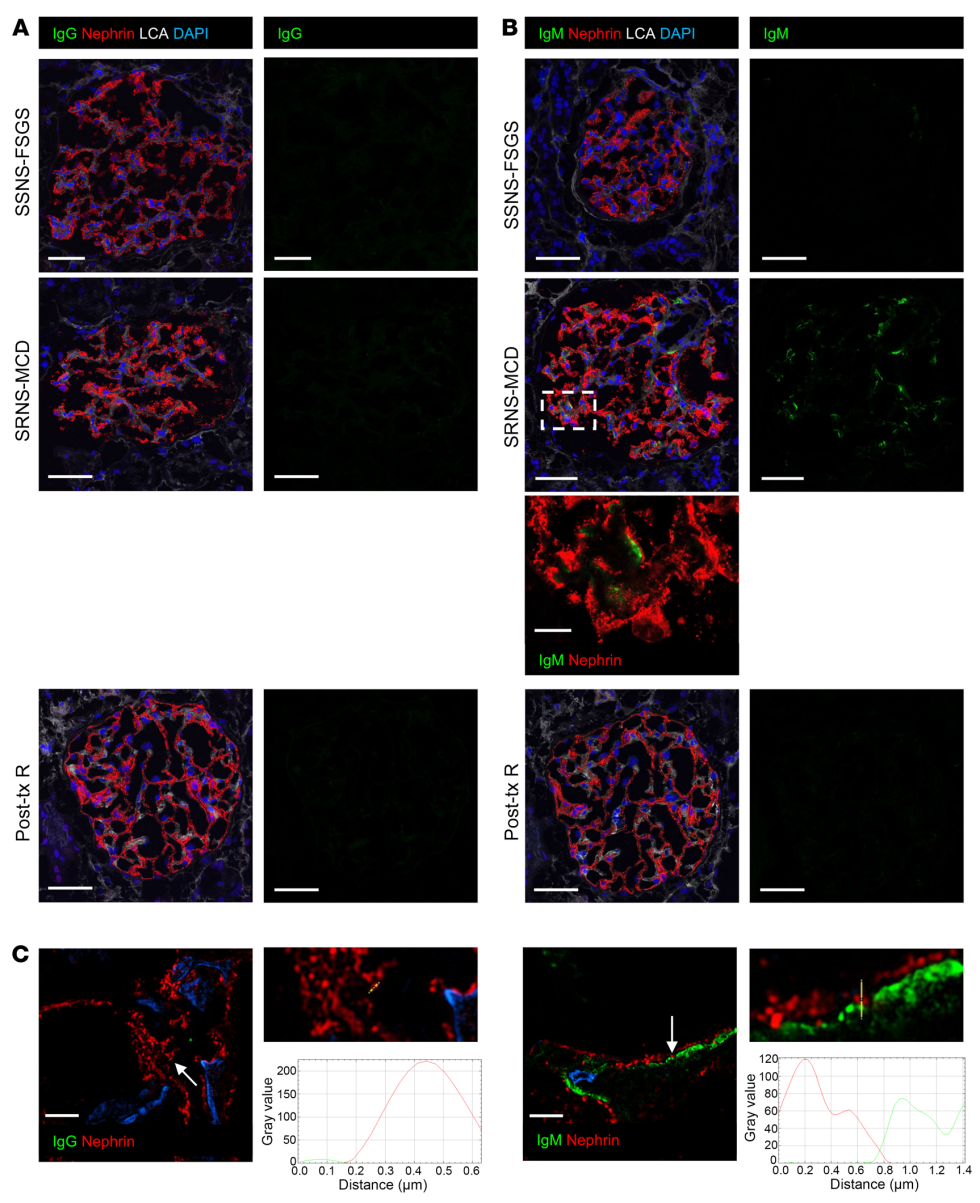
IgG, IgM, and nephrin immunolabeling in kidney biopsies. (**A**) Representative double immunofluorescence staining for IgG (green) and nephrin (red) in biopsies of patients with steroid-sensitive nephrotic syndrome–focal segmental glomerulosclerosis (SSNS-FSGS; upper panels, *n =* 2), steroid-resistant nephrotic syndrome–minimal change disease (SRNS-MCD, middle panels, *n =* 3), and posttransplant focal segmental glomerulosclerosis recurrence (Post-tx R; lower panels, *n =* 6). The respective single staining of IgG is shown on the right. Scale bars: 5 μm. (**B**) Representative double immunofluorescence staining for IgM (green) and nephrin (red) in the different patient groups and, to the right, the images of IgM alone. Nuclei are stained with DAPI (blue) and renal structures with LCA (white). The inset at the bottom of the panel highlights the absence of colocalization between potential traces of IgM and nephrin. Scale bars: 50 μm (inset: 10 μm). (**C**) Representative SIM microscopy images of IgG/IgM and nephrin staining in renal biopsies. The graphs show signal intensity and the absence of colocalization. The insets and arrows indicate the points at which the measurements were performed. Scale bars: 5 μm.

Given the absence of detectable circulating anti-nephrin IgG in patients with primary and posttransplant recurrent podocytopathies, we next examined whether anti-nephrin antibodies could be observed at the renal level. We analyzed residual frozen renal biopsies from patients with steroid-sensitive nephrotic syndrome (*n =* 2 focal segmental glomerulosclerosis and *n =* 3 minimal change disease) and patients with steroid-resistant nephrotic syndrome (*n =* 3) at disease onset. Additionally, 6 patients with posttransplant podocytopathy recurrence, collected at the time of disease recurrence (median 26 days posttransplant), were also analyzed. Consistent with previous findings, nephrin staining exhibited a discontinuous, punctate pattern in patients with podocytopathies, in contrast with control kidney tissue, which exhibited continuous and uniform staining along the glomerular tuft (Supplemental Figure 8). In this setting, immunofluorescence analysis revealed a complete lack of IgG in the glomeruli (Figure 4A). Given that a recent study suggested the possibility that anti-nephrin antibodies may be of the IgM isotype (12), we also examined this Ig class in kidney biopsies from our patients. IgM antibodies were barely detectable; when present, they appeared only as faint, scattered traces along the glomerular basement membrane and did not colocalize with nephrin staining (Figure 4B). To achieve higher resolution and more accurate localization of nephrin, IgG, and IgM, we employed structured illumination microscopy (SIM) on the same renal specimens. In this high-resolution setting, the signal intensity peaks of nephrin were consistently separated from those of IgG and IgM. This pattern further supports the lack of colocalization between nephrin and either IgG or IgM (Figure 4C).

## Discussion

In this study, we conducted an extensive assessment of circulating anti-nephrin antibodies across primary and posttransplant recurrent podocytopathies, as well as appropriate disease and healthy controls. In this setting, we were unable to detect disease-enriched anti-nephrin antibodies in any of these clinical conditions when using both ELISA and immunoprecipitation assays based on human-derived nephrin. These data challenge the notion that anti-nephrin antibodies have a broad pathogenic and diagnostic relevance in minimal change disease and focal segmental glomerulosclerosis (8–12) and, most notably, posttransplant disease recurrence (15, 16, 21).

A central insight from our study is the profound influence of antigen origin on assay specificity. When nephrin produced in murine NS0 cells was used as the ELISA substrate, we observed unexpectedly high apparent seropositivity across all cohorts, with the highest rates paradoxically seen in healthy individuals. To investigate this phenomenon, we considered several pieces of well-established published data: (a) recombinant proteins generated in murine NS0 cells carry abundant α-Gal epitopes (22); (b) humans lack α1,3-galactosyltransferase and therefore do not synthesize α-Gal (23); and (c) anti–α-Gal antibodies are among the most prevalent natural antibody specificities in humans, driven by continuous immune exposure to α-Gal–expressing commensal bacteria (19). Through complementary biochemical and serological approaches, we demonstrated that the reactivity observed in control subjects against murine cell–derived nephrin was not actual anti-nephrin autoimmunity, but rather it was mainly driven by nonspecific binding of natural anti–α-Gal IgG. However, we cannot exclude that additional factors may contribute to the observed reactivity in patients with underlying autoimmune conditions, as exemplified by reports of antibody responses directed against protein purification tags in systemic lupus erythematosus and type 1 diabetes (20).

When this confounder was eliminated by employing nephrin produced in human cells, apparent anti-nephrin reactivity was nearly abolished and restricted to isolated samples. In this refined analytical framework, immunoprecipitation confirmed the ELISA finding that anti-nephrin antibodies were rare and not enriched in native nephrotic syndromes nor in posttransplant recurrence compared with HVs. Further supporting this, confocal and high-resolution microscopy of kidney biopsies obtained at the time of recurrence showed no IgG deposition and no colocalization of immunoglobulins with nephrin. Taken together, these data do not support a major pathogenic or clinical role for anti-nephrin antibodies in idiopathic nephrotic syndrome and posttransplant podocytopathy recurrence as a category.

In this context, our findings differ from recent reports using human cell–derived nephrin. Watts et al. (8) and Hengel et al. (9) detected high prevalence (~40%) of anti-nephrin antibodies employing in-house–generated recombinant nephrin from human cells. In this setting, factors such as protein boundaries, purification strategies, and the expression system used may account for the observed discrepancies. More recently, different studies took advantage of a commercially available antigen, introducing a greater degree of construct homogeneity across different populations (24). Although using the same commercial antigen derived from human cells, Shu et al. (12) described substantially higher seroprevalence in idiopathic nephrotic syndrome (~30%–40%). Several methodological factors may account for the observed differences with our study. High antigen-coating concentrations, low secondary antibody dilutions, and readings approaching the upper limit of the ELISA, for example, could all possibly enhance low-affinity or polyreactive binding and potentially inflate apparent positivity. In contrast, our assays were optimized according to international bioanalytical validation standards and cross-validated by immunoprecipitation, minimizing the risk of nonspecific reactivity. Our results are consistent with more recent evaluations that integrated ELISA, immunoprecipitation, and cell-based imaging and similarly reported very low anti-nephrin reactivity in patients with idiopathic nephrotic syndrome and posttransplant recurrence, with prevalences of approximately 3% and 0%, respectively (20). Among patients with posttransplant recurrent podocytopathies, we did not observe any meaningful anti-nephrin seropositivity, irrespective of sampling time (pretransplant, at recurrence, or short and long term after transplantation, whether on dialysis or not). Despite the wide range in time from transplant to recurrence in our cohort, implying a biological heterogeneity, our data are very consistent with those from a cohort of 161 kidney transplant recipients, in which only 4.3% tested positive for anti-nephrin IgG, with no difference between patients with or without posttransplant recurrence (20).

Together, these findings highlight the importance of a harmonized and widely adopted methodology to accurately contextualize results obtained across different patient cohorts and to evaluate the pathogenic relevance, if any, of anti-nephrin antibodies. Our conclusions support the recent statement from the ERA Immunonephrology Working Group, which emphasizes that further data using standardized commercial assays for the detection of anti-nephrin antibodies are essential before anti-nephrin antibodies can be considered a standard diagnostic and prognostic tool (25).

An additional consideration concerns the biological nature of the few anti-nephrin antibodies that we detected using human-derived antigen. In our experimental setting, this signal reflected true antigen-specific binding rather than glycan-mediated cross-reactivity, as demonstrated by deglycosylation experiments. This reactivity may be explained by the fact that nephrin contains 8 extracellular Ig-like domains, and several lines of evidence indicate that circulating natural antibodies frequently recognize conserved structural motifs shared across proteins of the Ig superfamily. This phenomenon has been demonstrated in multiple systems: natural antibodies present in pooled normal IgG (IVIG) bind nonpolymorphic regions of HLA class I molecules, containing several Ig-like folds (26). Similarly, IVIG also contains natural IgG directed against the Ig-like extracellular domains of Fcγ receptors CD16 and CD32 (27). To give another example, natural IgG responses to CD25, another receptor with an extracellular Ig-like structure, have also been documented in healthy subjects (28). Notably, natural IgG has also been identified against ICAM-1, an adhesion molecule that shares key structural features with nephrin, including heavy glycosylation and the presence of multiple extracellular Ig-like domains (29). Paradoxically, these antibodies have been shown to be more abundant in healthy individuals than in patients with inflammatory or autoimmune conditions (29). Taken together, these observations suggest that the low-level anti-nephrin antibodies detected in our cohort may, at least in part, reflect the presence of naturally occurring immunoglobulins rather than a disease-specific autoimmune response. By contrast, anti-nephrin antibodies have also been documented in broader clinical contexts unrelated to podocytopathies, including recurrent urinary tract infections (30) and diabetes mellitus (31), conditions not typically associated with podocyte injury. These observations further support the notion that anti-nephrin reactivity may occur outside the context of podocyte-specific disorders and does not necessarily indicate pathogenic, disease-driving autoimmunity.

From a clinical perspective, the data presented here suggest that anti-nephrin antibody testing, in its current form, provides limited information for the evaluation of native and posttransplant recurrent podocytopathies as a category. In our cohort of patients, antibody prevalence was low, sporadic, and indistinguishable from healthy controls, with no relationship to disease activity or timing of recurrence. Together with the absence of glomerular Ig deposition, these results argue against incorporating anti-nephrin serology into diagnosis, monitoring protocols, or therapeutic guidance as a broadly applicable biomarker across podocytopathies. At the same time, our findings do not fully exclude the possibility that anti-nephrin antibodies may be present in rare or context-specific subsets of patients, potentially defined by factors such as disease stage, age, clinical phenotype, or antibody isotype. Future efforts should instead prioritize the identification of meaningful circulating factors and molecular signatures that more accurately drive podocyte injury and better inform personalized clinical management.

## Methods

### Sex as a biological variable.

Our study investigated data from both male and female human participants, and similar findings are reported for both sexes. Sex data are reported in Tables 1 and 2.

### Patient cohort.

Adult patients with primary podocytopathy and documented disease recurrence after transplantation were recruited from the Nephrology Unit at ASST-Papa Giovanni XXIII, the Clinical Research Center for Rare Diseases “Aldo e Cele Daccò” of the Istituto di Ricerche Farmacologiche Mario Negri IRCCS, and from the Nephrology, Dialysis, and Renal Transplantation Unit of the Policlinico Hospital in Milan. Age- and sex-matched HVs were also recruited. Serum samples from patients were obtained from the UNI EN ISO 9001–certified research biobank Centro di Risorse Biologiche Mario Negri–Biobanca Malattie Rare e Renali, established at the Centro Daccò of the Mario Negri Institute, or collected ad hoc. All adult patients were diagnosed by renal biopsy, except for 4 cases: 3 SSNS patients with pediatric-onset disease and 1 SSNS patient with a contraindication to biopsy due to a solitary kidney. Pathological lesions for the patients for whom the medical reports were available are reported in Table 3 (20/23 posttransplant podocytopathy recurrence, 13/16 steroid-resistant nephrotic syndrome, and 30/39 steroid-sensitive nephrotic syndrome). Active disease was defined as nephrotic-range proteinuria (>3.5 g/day) and serum albumin <3.5 mg/dL. Remission was defined as < 0.3 g/day protein excretion, along with normal serum albumin concentration (32).

Pediatric patients with posttransplant podocytopathy recurrence or steroid-resistant/multidrug-dependent nephrotic syndrome were recruited at Istituto Giannina Gaslini IRCCS, in accordance with the Regional Ethics Committee (CER Liguria: 115/2023, DB ID 13009). Informed consent was obtained from relatives for subjects < 18 years old. According to KDIGO guidelines, biopsy was not performed at disease onset in pediatric patients or during childhood. In these patients, diagnosis was made based on response to immunosuppressive treatment.

Race and ethnicity data were collected by investigators based on self-reported information from participants or their legal guardians, according to NIH categories. Among patients with steroid-sensitive nephrotic syndrome, 2 identified as Asian, 2 as Black or African American, and the remaining 35 as White. All pediatric patients and all participants in the other cohorts identified as White. Ethnicity data (Hispanic/Latino) were not systematically collected given the predominantly European origin of the study population.

### Optimized in-house ELISA for anti-nephrin antibody detection.

As a first step, we tested a commercially available anti-nephrin ELISA kit and a previously described anti-nephrin ELISA method (10, 15), but they failed to yield reliable data (Supplemental Methods). To overcome this limitation, we set up an optimized in-house ELISA. Microtiter 96-well plates (Life Technologies) were coated with 100 ng/well of the extracellular domain of recombinant human nephrin (UniProtKB, accession O60500) produced using a mouse myeloma cell line (R&D Systems, 9399-NN; domain Gln23-Thr1029) or human embryonic kidney cells (HEK293; SinoBiological, 17757-H08H; domain Met1-Ser1055). Both antigens were diluted in 1× PBS (Life Technologies). In selected experiments, antigens were treated with different deglycosidases or left untreated to assess glycan-dependent serum reactivity, as described in Supplemental Methods. In parallel, control wells were filled with 100 μL/well of 1× PBS to assess nonspecific binding background signals from serum samples. Plates were incubated overnight at 4°C. Plates were washed 4 times with 300 μL/well of wash buffer composed of 1× PBS, 0.1% Tween 20 (Fisher Scientific), and 0.5 M NaCl (Merck) and then blocked for 1 hour at room temperature (RT) with 200 μL/well of blocking buffer composed of 1× PBS, 0.1% Tween 20, and 0.5% skim milk (Merck). Plates were then incubated for 40 minutes at RT with 50 μL/well serum samples diluted at 1:50 in blocking buffer. A 7-point standard curve was generated only in the ELISA, using murine cell–derived nephrin as the coated antigen. Serial 2-fold dilutions of a high-absorbance reference serum were used (50 μL of each standard per well). The concentration was assigned arbitrarily to each standard, with a range between 2,000 and 31 AUs per milliliter.

After sera incubation, plates were washed 4 times with 300 μL/well of wash buffer and incubated for 1 hour at RT with 100 μL/well of HRP-conjugated goat anti-human IgG Fc secondary antibody (1:20,000; Invitrogen, A18817) in blocking buffer. We also tested a 1:4,000 secondary antibody dilution, the most frequently used concentration in published studies. Under this condition, wells without antigen coating exhibited optical density values exceeding 50% of those of coated wells, resulting in a signal-to-background ratio of <2. According to international assay validation guidelines (33, 34), such ratios do not meet reliability criteria for quantitative interpretation.

After incubation, plates were washed 6 times with 300 μL/well of wash buffer and incubated for 20 minutes with 100 μL/well of tetramethylbenzidine substrate (Thermo Fisher Scientific, N301). The colorimetric reaction was stopped by adding 100 μL/well of 2 N sulfuric acid solution (Thermo Fisher Scientific, 035610.K2). Values of OD_450_ were measured using an iMark Microplate Absorbance Reader (Bio-Rad Laboratories). For each sample, the absorbance value from uncoated wells was subtracted from nephrin-coated wells to account for nonspecific binding. All samples were run in duplicate. There was good linearity between absorbance values and serum dilution, and the absorbance values in the noncoated wells were negligible. In assays with murine cell–derived nephrin, the positivity threshold (180 AU/mL) was determined as the mean of the concentrations detected in the sera of HVs testing negative for anti–α-Gal antibodies plus 2 SDs to avoid the confounding effects of anti–α-Gal–mediated cross-reactivity. In assays with human cell–derived nephrin, only OD_450_ values were considered. Under our optimized conditions, uncoated wells showed a mean OD of approximately 0.090, below values reported in comparable studies (35). The positivity threshold (0.150 OD) was determined as the mean of the optical densities detected in all HV sera (excluding HV3, the positive sample), plus 2 SDs.

In both ELISAs, we tested a serial dilution (1:1,000, 1:2,000, 1:4,000 in blocking buffer) of a commercial sheep anti-human nephrin antibody (R&D Systems, AF4269) in parallel. For detection, HRP-conjugated donkey anti-sheep IgG secondary antibody (R&D Systems, HAF016) was used. In both ELISAs, anti-nephrin polyclonal antibody was detected with similar absorbance values for plates coated with nephrin from mouse and human cells (Supplemental Table 2). The dilution of anti-nephrin polyclonal antibody in human serum AB did not affect its detection, supporting the reliability of ELISAs for anti-nephrin antibody detection and indicating the absence of interfering factors in human serum.

### IP-WB analysis.

Immunoprecipitation was performed as previously described (8). Briefly, 3 μL of serum samples was incubated overnight at 4°C with 100 μL of Tris-buffered saline supplemented with 0.1% Tween-20 (TBSt) and 100 ng of recombinant nephrin produced in human embryonic kidney cells (HEK293; SinoBiological, catalog 17757-H08H). Following incubation, IgG-nephrin complexes were precipitated with 15 μL of protein G magnetic beads (Cytiva Protein G Mag Sepharose, 28951379) for 4 hours at RT. At the end of the incubation, samples were washed 5 times with 200 μL of TBSt. Proteins were eluted from the beads and denatured under reducing conditions by heating them at 96°C for 3 minutes in 1× Laemmli sample buffer (Bio-Rad Laboratories, 1610747) supplemented with 2.5% β-mercaptoethanol (Bio-Rad Laboratories, 1610710). Samples were separated on 4%–15% Mini-PROTEAN TGX Precast Protein Gels (Bio-Rad Laboratories, 4561084) and transferred to 0.2 μm nitrocellulose membranes (Bio-Rad Laboratories, 1704159). After blocking with 5% BSA in TBSt, membranes were incubated overnight at 4°C with sheep anti-human nephrin (1:800; R&D Systems, AF4269). The signals were visualized on an Odyssey FC Imaging System (LiCor) with an HRP-conjugated donkey anti-sheep IgG secondary antibody (1:4,000; Sigma Aldrich, A3415) followed by incubation with SuperSignal West Pico PLUS Chemiluminescent Substrate (Thermo Fisher Scientific, 34580).

Western blot analysis of α-Gal epitopes and enzymatic deglycosylation experiments are described in detail in Supplemental Methods.

### Immunohistochemical analysis of kidney biopsies.

Single and double immunofluorescence experiments were performed on kidney biopsies that were snap-frozen and embedded in OCT compound. Two-micrometer-thick cryosections were fixed in acetone for 10 minutes at 4°C and then hydrated. The sites of nonspecific binding were blocked with 1% BSA. Consecutive sections were incubated with sheep anti-human nephrin antibody (1:100; R&D Systems, AF4269) for 2 hours at RT, followed by the Cy3-conjugated rabbit anti-sheep IgG secondary antibody (1:100, Jackson ImmunoResearch Laboratories, 313-165-045) and then with FITC-conjugated rabbit anti-human IgG or IgM antibodies (1:50; DakoCytomation, F0202 and F0203, respectively) for 1 hour at RT. A negative control for nephrin immunostaining was obtained by omitting the primary antibody on the adjacent section. Nuclei were stained with DAPI, while Dylight 649 Lens Culinaris Agglutinin (LCA) was used to highlight renal structures. Fluorescence was examined with an inverted confocal laser microscope (Leica Microsystems, Leica SP8). The same sections were analyzed using super-resolution SIM (Supplemental Methods).

Under the same experimental conditions, a biopsy sample from a patient with MN was used as a positive technical control to validate the tissue-based immunofluorescence workflow. In this setting, IgG localization was assessed together with phospholipase A2 receptor (PLA2R), detected with a rabbit anti-PLA2R antibody (1:50; Sigma-Aldrich, HPA012657). This approach confirmed the ability of the technique to identify IgG antigen–specific immune deposits with a characteristic granular pattern (Supplemental Figure 9).

### Statistics.

Data are presented as frequency counts or percentages and as the mean ± SEM. Data were analyzed using unpaired 2-tailed *t* test or 1-way ANOVA coupled with Tukey’s post hoc analysis, as appropriate. Single data points are displayed as dot plots. The statistical significance level was defined as *P*  value < 0.05. Data analysis was performed using GraphPad Prism (GraphPad Software). Receiver operating characteristic curve analysis for anti–α-Gal ELISA was performed to determine the discriminative performance of the ELISA and to establish the optimal cutoff value for serum IgG antibodies against the α-Gal epitope. The optimal cutoff was identified according to the maximum Youden index (J = sensitivity + specificity − 1), providing the best balance between sensitivity and specificity. For paired measurements obtained from the same serum under different preabsorption conditions, differences were analyzed using Friedman’s test followed by Wilcoxon’s signed-rank tests with Bonferroni correction.

### Study approval.

The study protocols (PARSEC and BLINDER studies) were approved by the Comitato Etico Territoriale Lombardia 6 Ethics Committee, and all participants provided written informed consent. Pediatric patients with posttransplant podocytopathy recurrence or steroid-resistant/multidrug-dependent nephrotic syndrome were recruited at Istituto Giannina Gaslini IRCCS, in accordance with the Regional Ethics Committee (CER Liguria: 115/2023, DB ID 13009). Written informed consent was obtained from relatives for subjects < 18 years old. These studies were conducted in accordance with the principles outlined in the Declaration of Helsinki.

### Data availability.

All data supporting the findings of this study are available within the article and its supplemental material. The datasets presented in this study can be found in the online repository Zenodo (https://zenodo.org/records/17701268) and in the Supporting Data Values file.

## Author contributions

Conceptualization and study design: LP, FC, AB, and GR. Investigation and methodology: FP, LP, FC, PR, XK, AS, M Trillini, M Todeschini, and MM. Data interpretation: FP, LP, FC, PR, M Trillini, AB, and GR. Writing — first draft: FP, LP, and FC. Writing — review and editing: AA, MAP, GC, M Trillini, AB, and GR. Co–first authorship order was determined by mutual agreement reflecting equal contributions.

## Conflict of interest

The authors have declared that no conflict of interest exists.

## Funding support

- The Medici di Marignano family.

## Supplementary Material

Supplemental data

ICMJE disclosure forms

Unedited blot and gel images

Supporting data values

## Figures and Tables

**Table 1:**
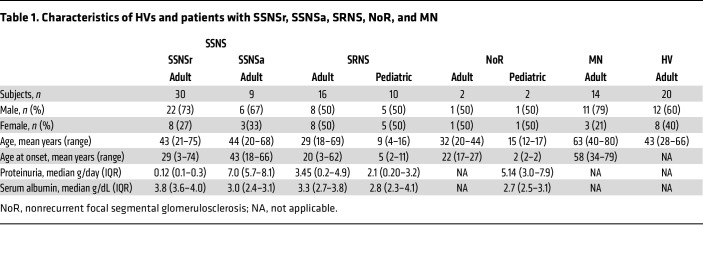
Characteristics of HVs and patients with SSNSr, SSNSa, SRNS, NoR, and MN

**Table 2:**
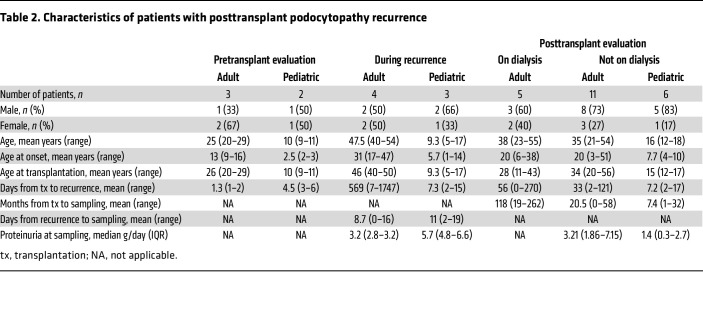
Characteristics of patients with posttransplant podocytopathy recurrence

**Table 3:**
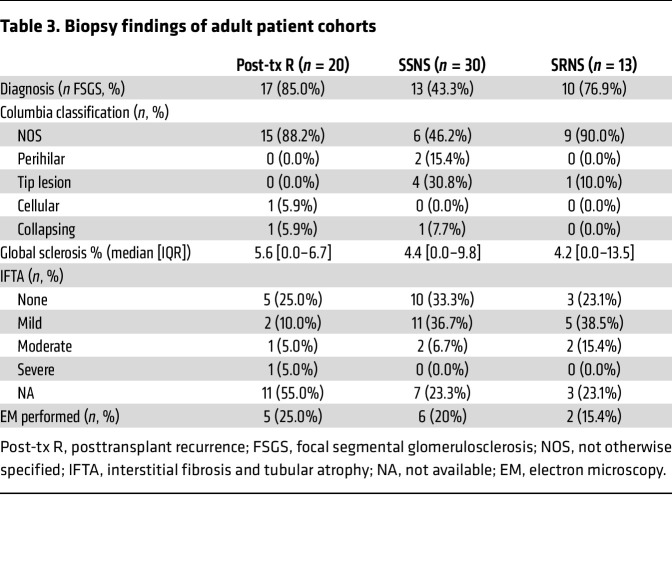
Biopsy findings of adult patient cohorts

## Version 1 04/28/2026

In-Press Preview

## Version 2 07/01/2026

Electronic publication

## References

[1] Kopp JB (2020). Podocytopathies. Nat Rev Dis Primers.

[2] Perico L (2016). Podocyte-actin dynamics in health and disease. Nat Rev Nephrol.

[3] Bonilla M (2024). A review of focal segmental glomerulosclerosis classification with a focus on genetic associations. Kidney Med.

[4] De Vriese AS (2021). Therapeutic trials in adult FSGS: lessons learned and the road forward. Nat Rev Nephrol.

[5] Raina R (2024). Post-transplant recurrence of focal segmental glomerular sclerosis: consensus statements. Kidney Int.

[6] Uffing A (2020). Recurrence of FSGS after kidney transplantation in adults. Clin J Am Soc Nephrol.

[7] Salfi G (2023). Current understanding of the molecular mechanisms of circulating permeability factor in focal segmental glomerulosclerosis. Front Immunol.

[8] Watts AJB (2022). Discovery of autoantibodies targeting nephrin in minimal change disease supports a novel autoimmune etiology. J Am Soc Nephrol.

[9] Hengel FE (2024). Autoantibodies targeting nephrin in podocytopathies. N Engl J Med.

[10] Raglianti V (2024). Anti-slit diaphragm antibodies on kidney biopsy identify pediatric patients with steroid-resistant nephrotic syndrome responsive to second-line immunosuppressants. Kidney Int.

[11] Chebotareva N (2024). A pilot study of anti-nephrin antibodies in podocytopaties among adults. Nephrology (Carlton).

[12] Shu Y (2025). Anti-nephrin antibodies in adult Chinese patients with minimal change disease and primary focal segmental glomerulosclerosis. Kidney Int.

[13] Bressendorff I (2024). Antinephrin-associated primary focal segmental glomerulosclerosis successfully treated with plasmapheresis. Kidney Int Rep.

[14] Hengel FE (2025). Anti-nephrin autoantibodies in steroid-resistant nephrotic syndrome may inform treatment strategy. Kidney Int.

[15] Shirai Y (2024). A multi-institutional study found a possible role of anti-nephrin antibodies in post-transplant focal segmental glomerulosclerosis recurrence. Kidney Int.

[16] Batal I (2024). Pre-transplant anti-nephrin antibodies are specific predictors of recurrent diffuse podocytopathy in the kidney allograft. Kidney Int.

[17] Kidney Disease: Improving Global Outcomes (KDIGO) Glomerular Diseases Work Group (2021). KDIGO 2021 clinical practice guideline for the management of glomerular diseases. Kidney Int.

[18] Yehuda S, Padler-Karavani V (2020). Glycosylated biotherapeutics: immunological effects of N-glycolylneuraminic acid. Front Immunol.

[19] Perusko M (2024). The α-Gal epitope - the cause of a global allergic disease. Front Immunol.

[20] Liu P (2025). Evaluation of methodologies in anti-nephrin autoantibody detection. Kidney Int.

[21] Shirai Y (2025). Reevaluating anti-nephrin autoantibodies by ELISA using human embryonic kidney-derived recombinant extracellular domain of human nephrin. Kidney Int.

[22] Yoo EM (2010). Differences in N-glycan structures found on recombinant IgA1 and IgA2 produced in murine myeloma and CHO cell lines. MAbs.

[23] Singh S (2021). Loss of α-gal during primate evolution enhanced antibody-effector function and resistance to bacterial sepsis. Cell Host Microbe.

[24] Wang J (2026). Association between anti-nephrin antibodies and podocytopathies: a systematic review and meta-analysis. Nephron.

[25] Mirioglu S (2025). Quo Vadis standardization of anti-nephrin antibody detection?. Glomerular Dis.

[26] Kaveri S (1996). Antibodies to a conserved region of HLA class I molecules, capable of modulating CD8 T cell-mediated function, are present in pooled normal immunoglobulin for therapeutic use. J Clin Invest.

[27] Bouhlal H (2014). Natural autoantibodies to Fcγ receptors in intravenous immunoglobulins. J Clin Immunol.

[28] Zhao H (2018). Alteration of circulating natural autoantibodies to CD25-derived peptide antigens and FOXP3 in non-small cell lung cancer. Sci Rep.

[29] Herman ML (2025). ICAM-1 autoantibodies detected in healthy individuals and cross-react with functional epitopes. Immunohorizons.

[30] Bruschi M (2024). Autoantibodies targeting nephrin in podocytopathies. N Engl J Med.

[31] Aaltonen P (2007). Circulating antibodies to nephrin in patients with type 1 diabetes. Nephrol Dial Transplant.

[32] Rovin BH (2021). Executive summary of the KDIGO 2021 guideline for the management of glomerular diseases. Kidney Int.

[33] https://www.fda.gov/regulatory-information/search-fda-guidance-documents/bioanalytical-method-validation-guidance-industry.

[34] https://www.ema.europa.eu/en/ich-q2r2-validation-analytical-procedures-scientific-guideline.

[35] Bianchi G (2025). Detection of antinephrin antibodies in childhood idiopathic nephrotic syndrome. Kidney Int Rep.

