# Supplementary material for: Anti-nephrin antibodies are not enriched in patients with primary and posttransplant recurrent podocytopathies
Source: J Clin Invest. 2026 Apr 28;136(13):e204727. doi: 10.1172/JCI204727 (PMC13318109; doi:10.1172/JCI204727)
Supplement: Supplemental data [file jci-136-204727-s150.pdf]

Supplemental Material for

Anti-nephrin antibodies are not enriched in patients with primary and post-transplant recurrent podocytopathies

Francesco Pecoraro[1*], Luca Perico[1*], Federica Casiraghi[1], Paola Rizzo[1], Matias Trillini[2], Andrea Angeletti[3], Manuel Alfredo Podestà[4], Xhuliana Kajana[3], Agnese Spennacchio[3], Marta Todeschini[1], Marilena Mister[1], Giuseppe Castellano[4], Ariela Benigni[1°], Giuseppe Remuzzi[1,2]

[1]Department of Molecular Medicine, Istituto di Ricerche Farmacologiche Mario Negri IRCCS, Bergamo, Italy; [2]Clinical Research Center for Rare Diseases "Aldo e Cele Daccò", Istituto di Ricerche Farmacologiche Mario Negri IRCCS, Bergamo, Italy; [3]Division of Nephrology, Dialysis, Transplantation, Istituto Giannina Gaslini IRCCS, Genoa, Italy.; [4]Unit of Nephrology, Dialysis and Renal Transplantation, Fondazione IRCCS Ca' Granda Ospedale Policlinico, Milan, Italy.

* Contributed equally as first authors

°Corresponding author:
ariela.benigni@marionegri.it

This file includes:

- Supplemental Methods
- Supplemental References
- Supplemental Table 1
- Supplemental Table 2
- Supplemental Figure 1
- Supplemental Figure 2
- Supplemental Figure 3
- Supplemental Figure 4
- Supplemental Figure 5
- Supplemental Figure 6
- Supplemental Figure 7
- Supplemental Figure 8
- Supplemental Figure 9

Supplemental methods

Commercial and in-house ELISA for anti-nephrin antibody detection

Serum samples from 3 patients with post-transplant podocytopathies recurrence, 3 membranous nephropathy patients, 3 healthy volunteers and a commercial AB serum preparation (pooled human serum AB, male, Euroclone) were tested at two concentrations (1:2 as suggested by the manufacturer's instruction and 1:100) to evaluate the levels of anti-nephrin antibodies by a commercial ELISA kit (Abbexa). Results showed an excellent standard curve (r^2=1) but unreliable results. There was no correlation between concentration of anti-nephrin antibodies and sample dilution. Samples diluted 1:100 gave very high concentration values and absorbance values higher than serum samples diluted 1:2. Among these samples, post-transplant podocytopathy recurrent patients and membranous nephropathy patients had similar anti-nephrin concentrations, which were below the detection limit with 1:2 dilution and about 200 and 250 ng/mL with the dilution 1:100, respectively. The highest levels of anti-nephrin antibodies were observed in healthy volunteers. The concentration of anti-nephrin antibodies in human serum AB was 5.22 ng/mL (1:2) and 520 ng/mL (1:100). Based on these data we stopped using this kit to determine the concentration of anti-nephrin antibodies and set up the in-house ELISA test, as previously described (1–3).

We set up an in-house ELISA using commercial recombinant extracellular domain of human nephrin (R&D, BioTechne), as described (2, 3). Serum samples from 3 patients with post-transplant podocytopathy recurrence, 3 membranous nephropathy patients, 3 healthy volunteers and commercial AB serum preparation (pooled human serum AB male), measured using the commercial kit, were tested at a dilution of 1:100 and 1:400. We observed a correlation between absorbance values and sample dilution. Also with this method, healthy volunteers produced the highest values of absorbance (including AB serum), and similar values between post-transplant podocytopathy recurrent patients and membranous nephropathy patients. However, the absorbance values in non-coated wells were very high, suggesting unspecific binding of the secondary antibody. This unspecific binding did not improve with the addition of goat serum to the blocking buffer, or when we used a secondary antibody

directly conjugated with HRP but decreased significantly when skim milk was added to the blocking solution.

In-house ELISA for anti-α-Gal antibody detection

Serum IgG antibodies against the α-Gal epitope were quantified by an in-house ELISA (Bethyl Laboratories) as previously described,(4, 5) with minor modifications. Briefly, 96–well plates were coated overnight at 4 °C with α1-3Galβ1-4Glc-BSA (Dextra Laboratories, NGP0330) at a concentration of 2 µg/mL in 0.1 M carbonate buffer. Control wells were coated with PBS only. After washing, wells were blocked for 30 min at RT using Bethyl blocking buffer. Serum samples were diluted 1:100 and incubated for 90 min at 37 °C and antibodies were detected using goat anti-human IgG HRP-conjugated antibody (1:20,000; Invitrogen, A18817) for 1 h at RT. Measurement of O.D. was performed on the multimode microplate reader TECAN Infinite M200® PRO at 450 nm with a reference wavelength of 650 nm. To provide quantitative data, a standard curve was generated using a purified human anti-α-Gal IgG1 monoclonal antibody (Absolute Antibody, clone m86; Ab00532-10.0) serially diluted from 1000 ng/mL to 6.25 ng/mL. Results were expressed as µg/mL.

In selected experiments, diluted sera from healthy volunteers were pre-absorbed overnight with 10 µg BSA, 5 µg α1-3Galβ1-4Glc-BSA or 10 µg α1-3Galβ1-4Glc-BSA (Dextra Laboratories).

Western Blot analysis of α-Gal epitopes and antibody reactivity

For the analysis of α-Gal residues, 1 µg of nephrin derived from murine or human cells, 1 µg of α1-3Galβ1-4Glc-BSA (Dextra Laboratories), or 1 µg of BSA (Sigma Aldrich) were loaded onto the gel and transferred. After blocking, membranes were incubated with a mouse anti–α-Gal antibody (1:1.000; Absolute Antibody, clone m86, Ab00532-1.1) and a rabbit anti-human nephrin antibody (1:1.000; Abcam, clone EPTG3, ab80298). The signals were visualized on the Odyssey FC Imaging System (LiCor) by infrared (IR) fluorescence using a donkey anti-rabbit IRDye 680LT (1:1,000; LiCor, 926-68023), a goat anti-mouse IRDye 800CW (1:1,000; LiCor, 926-32210) secondary

antibody, as appropriate. In selected experiments, membranes were incubated with healthy volunteer sera (1:100) and detection was performed with a goat anti-human IgG HRP conjugate (1:20,000; Invitrogen, A18817) followed by incubation with SuperSignal™ West Pico PLUS Chemiluminescent Substrate (Thermo Fisher Scientific, 34580).

Enzymatic removal of glycans from recombinant nephrin

Recombinant nephrin produced in mouse cells (R&D Systems) and human cells (Sino Biological) was subjected to enzymatic deglycosylation using either the Enzymatic Protein Deglycosylation Kit (Sigma-Aldrich, EDEGLY) or α1-3,4,6-galactosidase (New England Biolabs, P0747S).

Briefly, 5 μg of recombinant protein was incubated with 1 μg of distinct deglycosylases in the appropriate reaction buffer under either denaturing or non-denaturing conditions. For denaturing conditions, samples were first denatured as recommended by the manufacturer prior to enzymatic digestion. To optimize enzymatic activity, digestions were performed under different conditions, including 1 hour at 37°C, 3 hours at 37°C, or 2 hours at room temperature followed by overnight incubation at 4°C. Following treatment, proteins were used for Western blot analysis to assess α-Gal epitopes and for in-house ELISA to evaluate anti-nephrin antibody binding.

Super-Resolution microscopy

Structured illumination microscopy (SIM) on histological sections was performed using a Nikon SIM system with a 100x 1.49 NA oil immersion objective, managed by NIS elements software. Tissues were imaged at laser excitation of 405 (for nuclei), 488 (for IgG or IgM) and 561 nm (for nephrin) with a 3D-SIM acquisition protocol. Fourteen-bit images sized 1024x1024 pixels with a single pixel of 0.030 μm were acquired in a gray level range of 0-4000 to exploit the linear range of the camera (iXon ultra-DU-897U, Andor) and to avoid saturation. Raw and reconstructed images were validated with the SIMcheck plugin of ImageJ (https://pubmed.ncbi.nlm.nih.gov/26525406/). Briefly, raw IgG images displayed a channel intensity profile (CIP) with a total variation of 9.6% and a modulation

contrast (MCN) of 4.4. Raw IgM images had a CIP of 8.8% and a MCN of 9.1; raw nephrin images a CIP of 9.9% and a MCN of 4.8. Thus, all acquisitions fell within an acceptable range of light collection. Motion and illumination variation (MIV) was satisfactory for all markers, with a prevalence of the white color indicating no major image drift across the three angles of grating rotation. The raw data for Fourier projections showed that the first order spots were clean for IgG and nephrin, blurred for IgM, and the second order spots visible but blurred for nephrin. Overall, the SIM set up was deemed to be satisfactorily calibrated, the acquisition parameters properly set and the sample correctly prepared. The analysis of the reconstructed image showed that the light was collected according to the typical 'flower' pattern of SIM. The inflection points in the radial profile plot made it possible to determine the effective resolution achieved after data reconstruction, which reached ≈142 nm for IgG, ≈131 nm for IgM and ≈146 nm for nephrin.

Statistics

Receiver operating characteristic (ROC) curve analysis for anti-α-Gal ELISA was performed using GraphPad Prism to determine the discriminative performance of the ELISA and to establish the optimal cut-off value for serum IgG antibodies against the α-Gal epitope. The optimal cut-off was identified according to the maximum Youden index ($J = \text{sensitivity} + \text{specificity} - 1$), providing the best balance between sensitivity and specificity. For paired measurements obtained from the same serum under different pre-absorption conditions, differences were analyzed using Friedman's test followed by Wilcoxon signed-rank tests with Bonferroni correction.

Supplemental references

1. Watts AJB, et al. Discovery of Autoantibodies Targeting Nephrin in Minimal Change Disease Supports a Novel Autoimmune Etiology. *J Am Soc Nephrol*. 2022;33(1):238–252.

2. Raglianti V, et al. Anti-slit diaphragm antibodies on kidney biopsy identify pediatric patients with steroid-resistant nephrotic syndrome responsive to second-line immunosuppressants. *Kidney Int*. 2024;106(6):1124–1134.

3. Shirai Y, et al. A multi-institutional study found a possible role of anti-nephrin antibodies in post-transplant focal segmental glomerulosclerosis recurrence. *Kidney Int*. 2024;105(3):608–617.

4. Joral A, et al. The Quantification of IgG Specific to α-Gal Could Be Used as a Risk Marker for Suffering Mammalian Meat Allergy. *Foods*. 2022;11(3):466.

5. Ailsworth SM, et al. IgG to Galactose-Alpha-1,3-Galactose: Impact of Alpha-Gal IgE Sensitization, Blood Type, and Tick Bites. *Antibodies*. 2025;14(2):43.

Supplemental Table 1. Ongoing immunosuppressive therapy at time of blood collection in non-dialysis patients with post-transplant podocytopathies recurrence.

	During recurrence		Post-transplant evaluation Not on dialysis	
	adult	pediatric	adult	pediatric
Number of patients, n	4	3	11	6
On double immunosuppression (%)	1 (25)	0 (0)	5 (45)	0 (0)
On triple immunosuppression (%)	2 (50)	3 (100)	6 (55)	6 (100)
On steroids (%)	2 (50)	3 (100)	10 (91)	6 (100)
Steroid dose, range (mg/day)	5-15	25-40	4-10	5-25
On CsA (%)	0 (0)	0 (0)	4 (36)	0 (0)
CsA dose, range (mg/day)	/	/	125-375	/
On Tac (%)	3 (75)	3 (100)	7 (64)	6 (100)
Tac dose, range (mg/day)	2-6	6-6.6	3-10	3.7-8.4
On MMF (%)	2 (50)	3 (100)	6 (55)	6 (100)
MMF dose, range (mg/day)	1000-1440	500-2000	500-2000	1500-2000
On AZA (%)	0 (0)	0 (0)	2 (18)	0 (0)
AZA dose, mg/day	/	/	50	/

AZA: azathioprine, CsA: cyclosporine A, MMF: Mycophenolate mofetil, Tac: tacrolimus

Supplemental Table 2. Absorbance values of two-fold dilution of sheep anti-human nephrin antibody in PBS or AB serum in ELISA plates coated with either R&D or SinoBiological Nephrin. Blank wells were incubated with PBS or AB serum in the absence of anti-nephrin antibody.

**Absorbance values (OD)**				
Antibody dilution	Nephrin R&D		Nephrin SB	
Dilution solution	*PBS*	*AB serum*	*PBS*	*AB serum*
1:1000	2.866	2.795	3.202	2.99
1:2000	1.874	1.712	2.161	2.112
1:4000	1.135	1.075	1.326	1.364
Blank (PBS/serum)	0	0	0	0

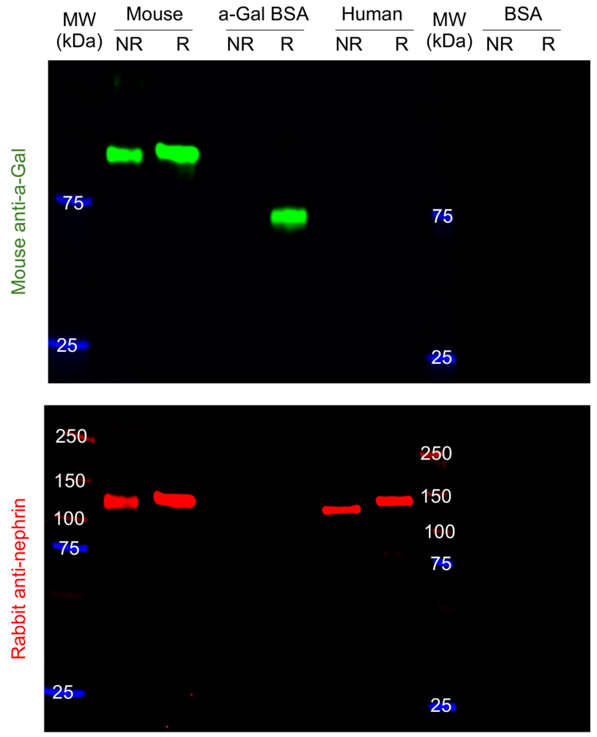

Supplemental Figure 1. Western blot analysis of recombinant human nephrin produced in murine cells (mouse), α1-3Galβ1-4Glc-BSA (α-Gal–BSA), recombinant human nephrin produced in human cells (human), and unconjugated BSA (BSA) under non-reducing (NR) and reducing (R) conditions. Membranes were probed with mouse anti–α-Gal antibody (green) and rabbit anti-nephrin antibody (red) to assess α-Gal modification. For each panel, molecular weights (MW) are expressed in Kilodalton (kDa).

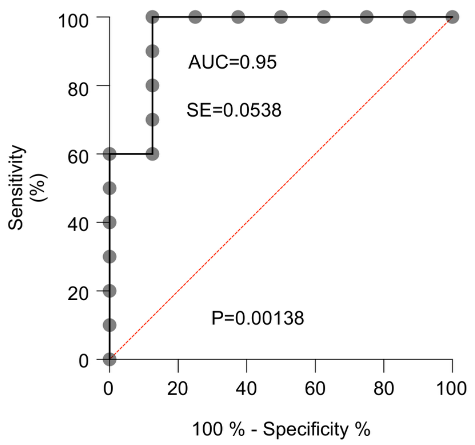

Supplemental Figure 2. Receiver operating characteristic (ROC) curve evaluating the discriminatory performance of the α-Gal antibody ELISA assay. The curve shows excellent diagnostic accuracy with an area under the curve (AUC) of 0.95 with a standard error (SE) of 0.0538 in distinguishing anti-nephrin–positive from anti-nephrin–negative sera. Statistical significance was assessed using the ROC comparison test (*P* value = 0.00138). The red diagonal line represents the reference line for a non-informative classifier.

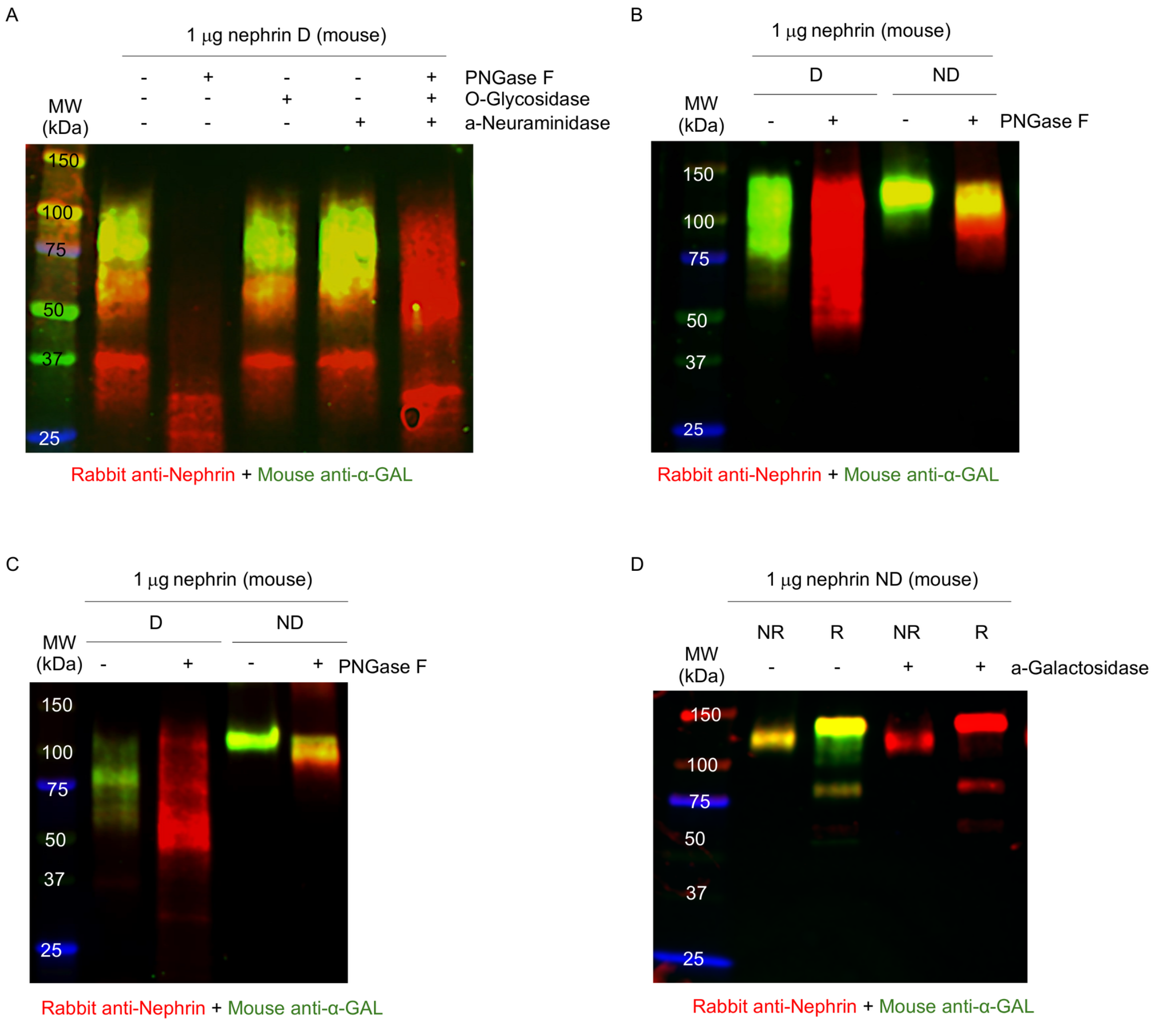

Supplemental Figure 3. (**A-C**) Western blot analysis of nephrin produced in mouse cells treated (+) or not (-) with PNGase F, O-glycosidase and α-neuraminidase in denaturing (D) or non-denaturing (ND) conditions for (**A**) 3 hours at 37°C, (**B**) 1 hour at 37°C or (**C**) 2 hours at room temperature followed by overnight at 4°C. (**D**) Representative Western blot analysis of nephrin derived from mouse cells treated (+) or not (-) with α-galactosidase in non-denaturing (ND) conditions and loaded under non-reducing (NR) or reducing (R) conditions. Membranes were probed with rabbit anti-nephrin antibody (red) and mouse anti–α-Gal antibody (green) to assess the presence of α-Gal epitopes. Molecular weights (MW) are expressed in Kilodalton (kDa).

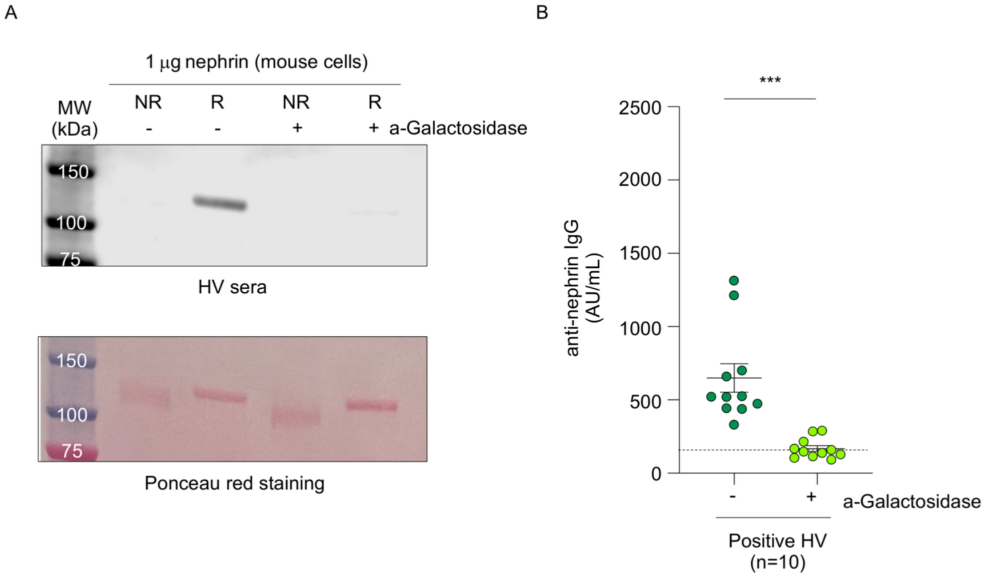

Supplemental Figure 4. (**A**) Western blot analysis of recombinant human nephrin produced in murine cells treated (+) or not (-) with α1-3,4,6 Galactosidase under non-reducing (NR) and reducing (R) conditions. Membranes were probed with sera from healthy volunteers (HV). Ponceau red staining is provided to assess proper protein loading, while molecular weights (MW) are expressed in Kilodalton (kDa). (**B**) ELISA assay with recombinant human nephrin produced in murine cells treated (+) or not (-) with α1-3,4,6-Galactosidase as coating antigen and performed on HV sera previously identified as anti-nephrin positive by ELISA with mouse cell-derived nephrin. The dotted line indicates the threshold for anti-nephrin antibody positivity, defined as 180 AU/mL. Data are mean ± SEM and analyzed using paired t-test. ****P* value <0.001.

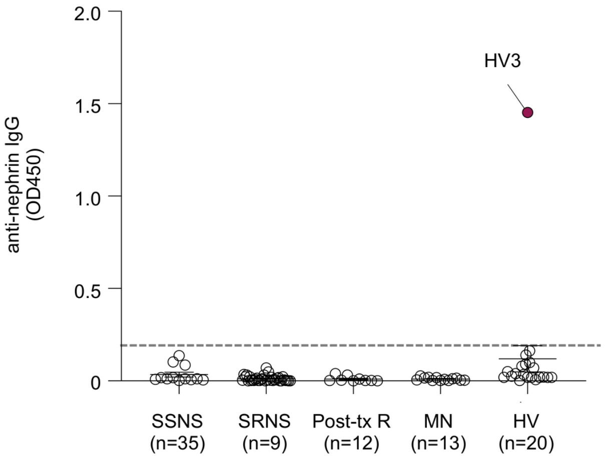

Supplemental Figure 5. Absorbance values at 450 nm (OD450) measured by ELISA using plates coated with the extracellular domain of recombinant human nephrin produced in human cells. Shown are the values from the same subjects analyzed with the ELISA employing human nephrin produced in mouse cells presented in Figure 1A. The dotted line indicates the threshold for anti-nephrin antibody positivity, defined as 0.150 OD. Data are mean ± SEM and analyzed using one-way ANOVA with Tukey's multiple comparison test. Post-tx R: post-transplant recurrence; SSNS: steroid-sensitive nephrotic syndrome; SRNS: steroid-resistant nephrotic syndrome; MN: membranous nephropathy; HV: healthy volunteers.

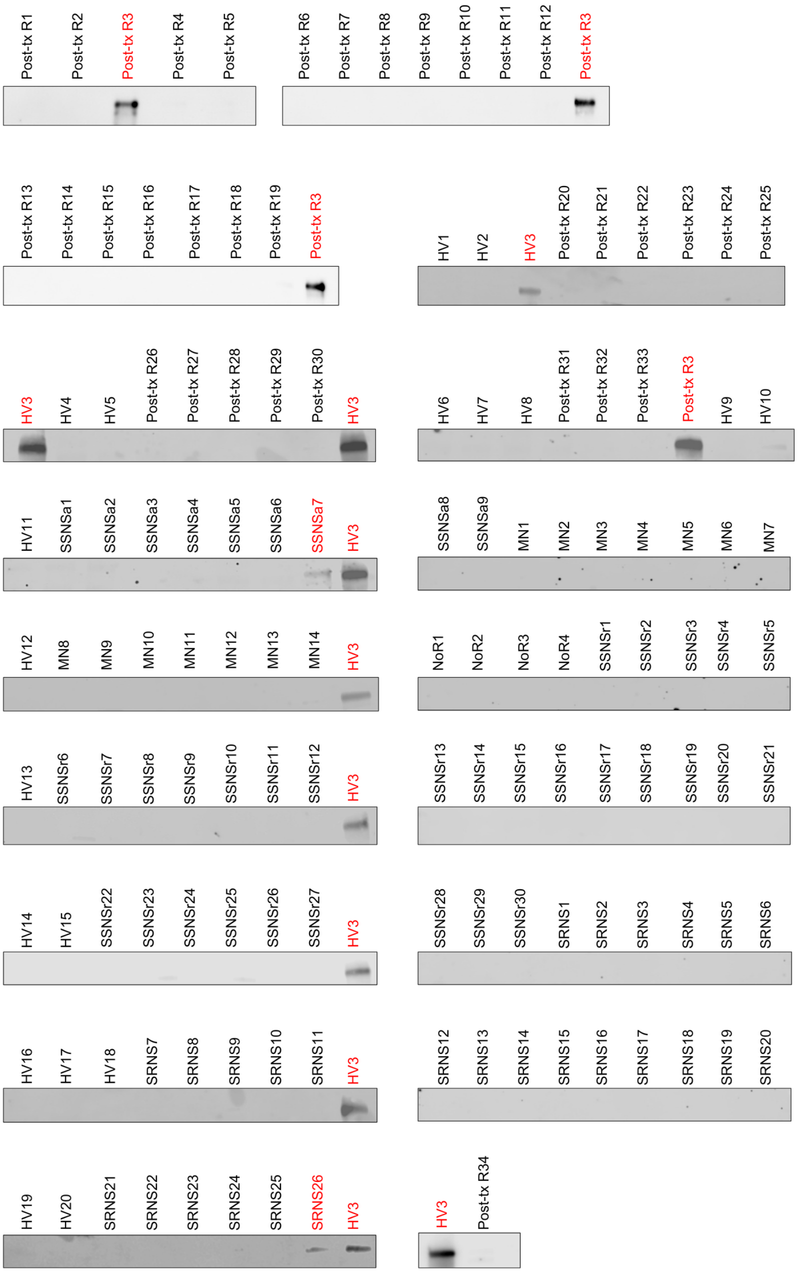

Supplemental Figure 6. Western blots after immunoprecipitation with recombinant human nephrin produced in human cells, using sera from the entire cohort. The dataset includes patients with post-transplant recurrence (Post-tx R, n=34), steroid-sensitive nephrotic syndrome in remission (SSNSr, n=30) or active disease (SSNSa, n=9), steroid-resistant nephrotic syndrome (SRNS, n=26), and non-recurrent transplant patients (NoR, n=4). Patients with membranous nephropathy (MN, n=14) and healthy volunteers (HV, n=20) served as controls. Detection was carried out using a sheep anti-nephrin primary antibody. Two strongly positive sera (HV3 and post-tx R3) were included in all Western blot experiments as internal intra- and inter-blot controls to ensure reproducibility and accuracy.

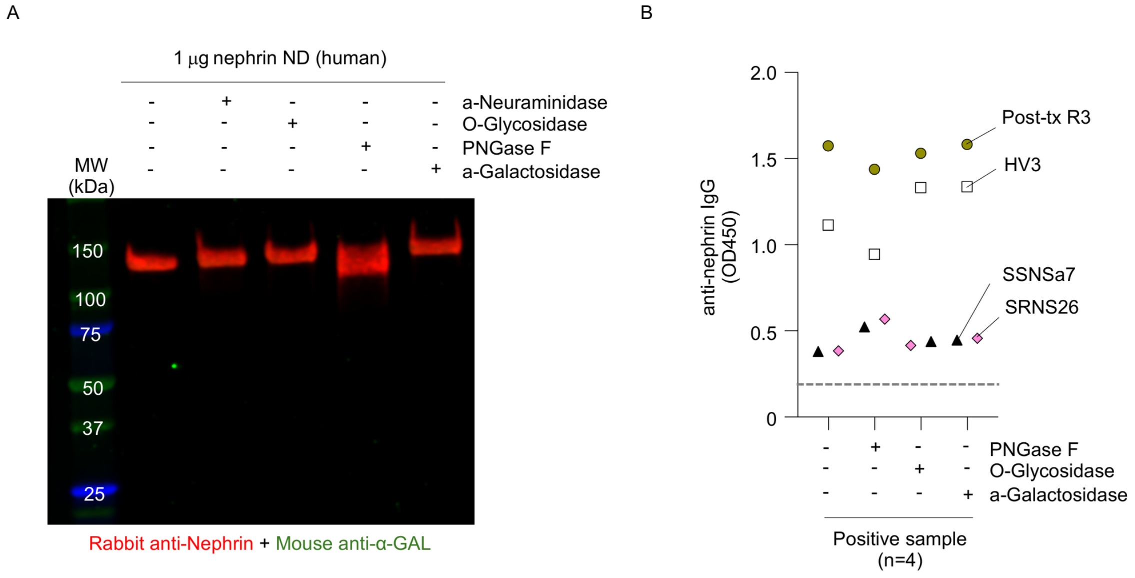

Supplemental Figure 7. (**A**) Western blot analysis of recombinant human nephrin produced in human cells treated (+) or not (-) with α-neuraminidase, O-glycosidase, PNGase F and α-galactosidase under non-denaturing (ND) conditions for 1h at 37°C. Membranes were probed with rabbit anti-nephrin antibody (red) and mouse anti–α-Gal antibody (green) to assess the presence of α-Gal epitopes. Molecular weight (MW) are expressed in Kilodalton (kDa). (**B**) ELISA assay with human cell-derived nephrin treated (+) or not (-) with PNGase F, O-glycosidase or α-galactosidase as coating antigen and performed on the 4 serum samples previously identified as anti-nephrin positive by ELISA with human cell-derived nephrin. The dotted line indicates the threshold for anti-nephrin antibody positivity, defined as 0.150 OD.

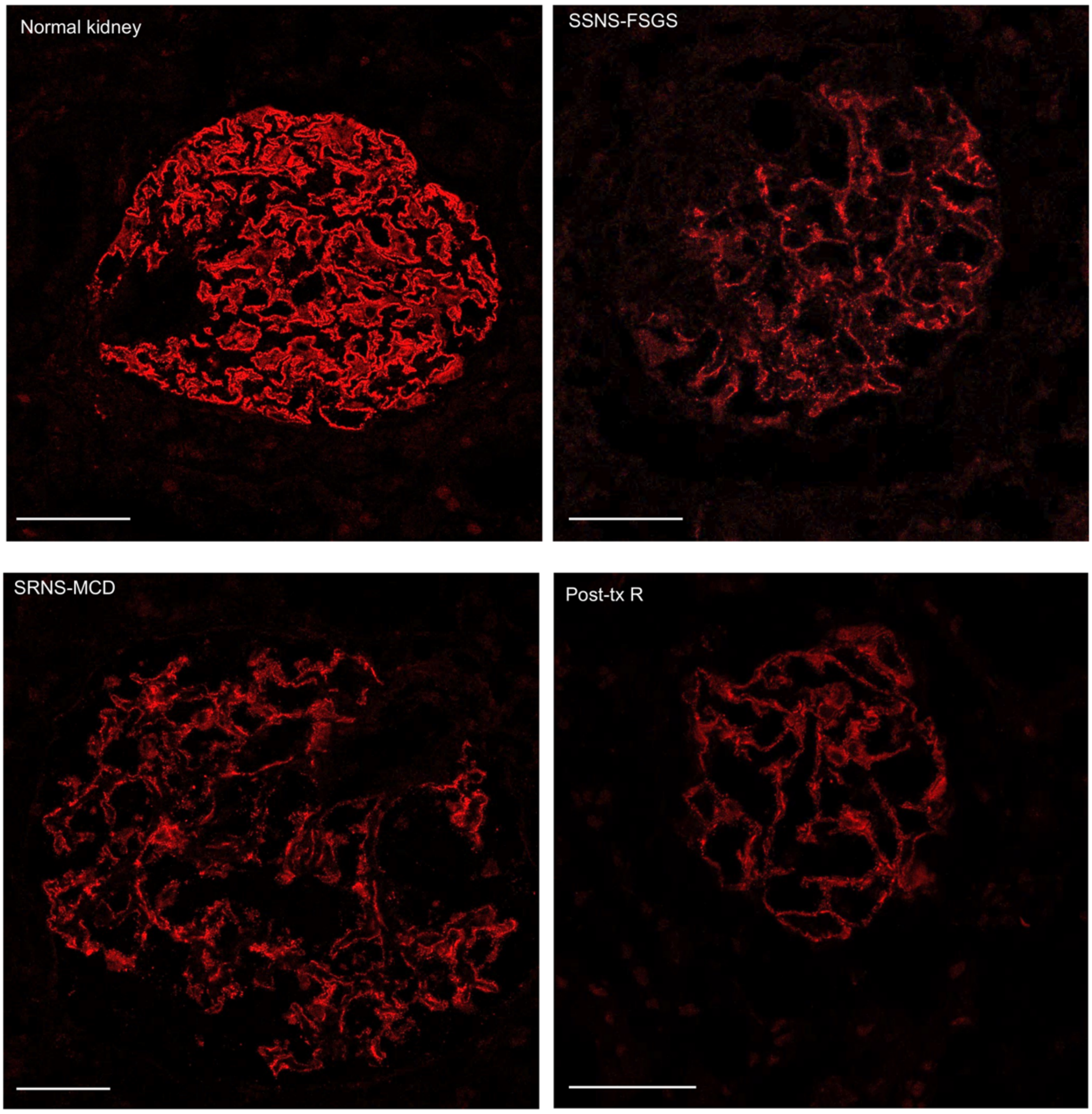

Supplemental Figure 8. Representative images of nephrin immunofluorescence staining (red) in normal kidney and in biopsies of patients with steroid-sensitive nephrotic syndrome with renal biopsy findings consistent with focal segmental glomerulosclerosis (SSNS-FSGS), steroid-resistant nephrotic syndrome with a minimal change disease pattern (SRNS-MCD), as well as patient with post-transplant FSGS recurrence (Post-tx R). Scale bar: 50 μm.

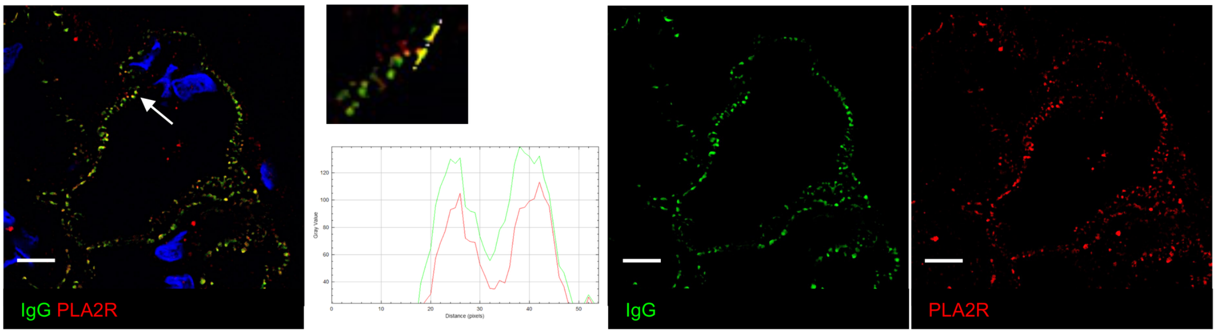

Supplemental Figure 9. Representative immunofluorescence staining for IgG (green) and Phospholipase A2 Receptor (PLA2R, red) in the renal biopsy of a patient with membranous nephropathy (MN). DAPI (blue) stained nuclei. The graphs show signal intensity and complete colocalization. The insets and arrows indicate the exact points in which the measurements were performed. The respective single stainings of IgG and PLA2R are shown on the right. Scale bars: 5 µm.
