# Supplementary material for: Anti-nephrin antibodies are not enriched in patients with primary and posttransplant recurrent podocytopathies
Source: J Clin Invest. 2026 Apr 28;136(13):e204727. doi: 10.1172/JCI204727 (PMC13318109; doi:10.1172/JCI204727)

Uncropped gel Figure 1B

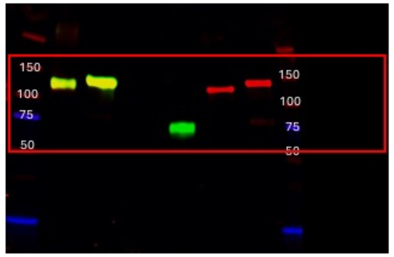

Uncropped gel Figure 1C

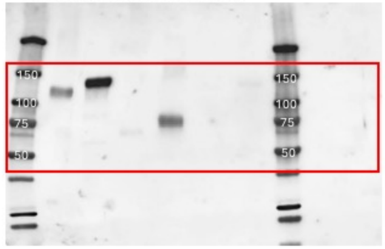

Uncropped gel Figure 2B

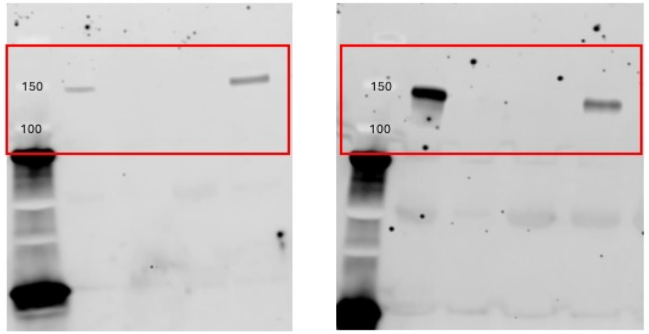

Uncropped gel Supplemental Figure 1

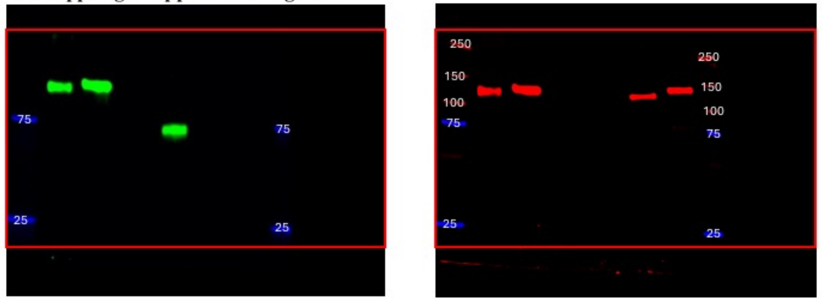

Uncropped gel Supplemental Figure 3A

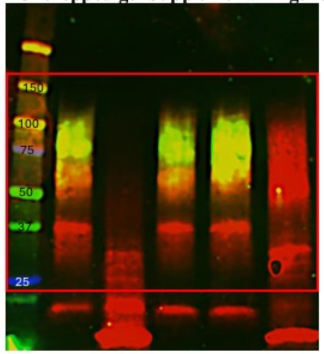

Uncropped gel Supplemental Figure 3B

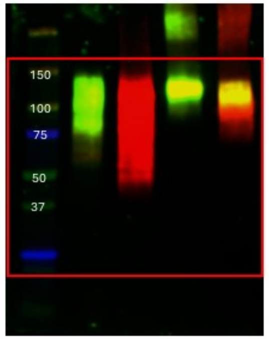

Uncropped gel Supplemental Figure 3C

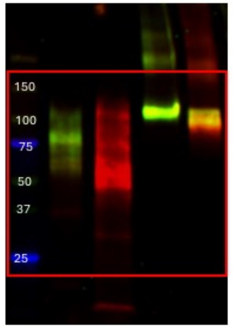

Uncropped gel Supplemental Figure 3D

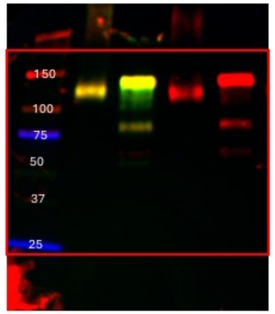

Uncropped gel Supplemental Figure 4A

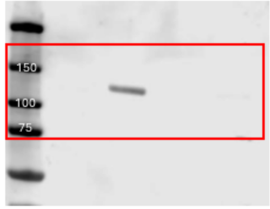

Uncropped gel Supplemental Figure 7A

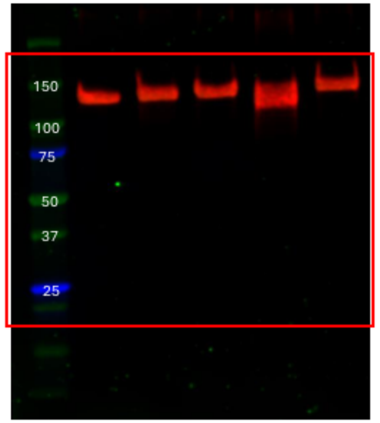

Uncropped gel Supplemental Figure 6

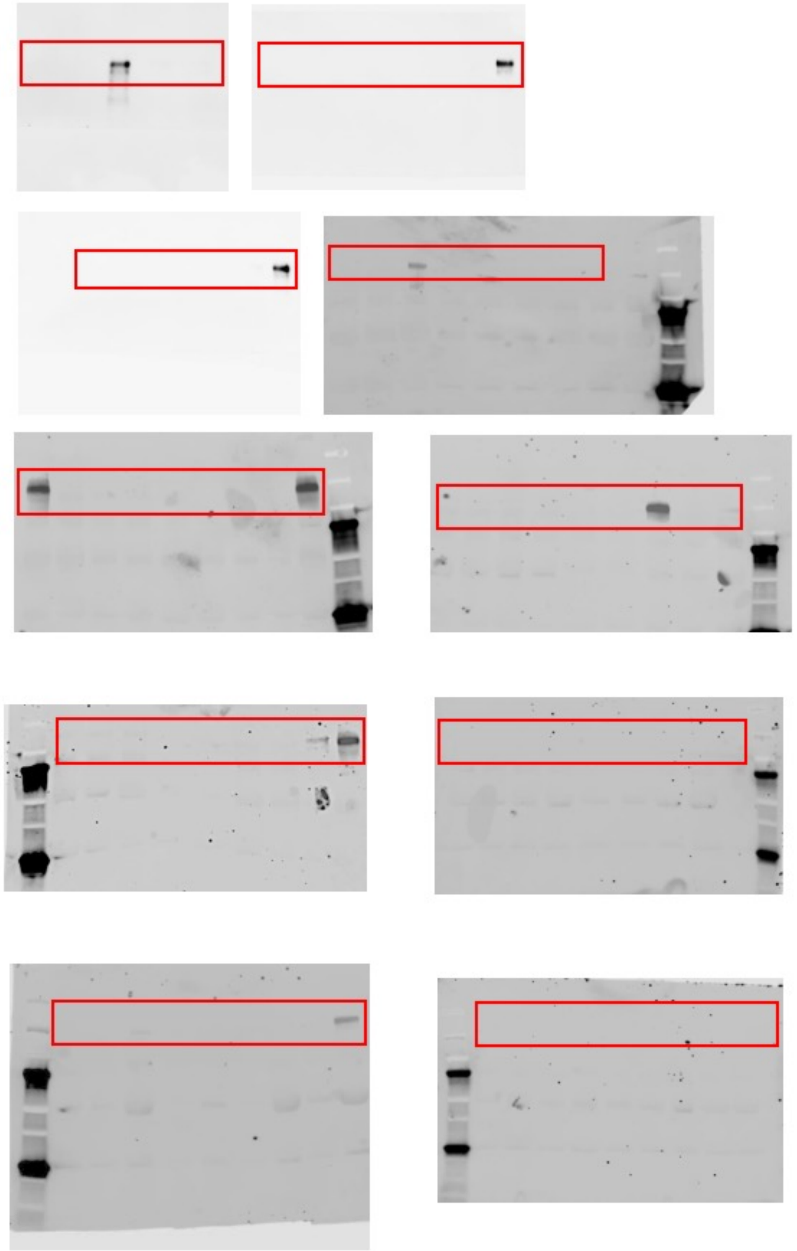

Uncropped gel Supplemental Figure 6 (continue)

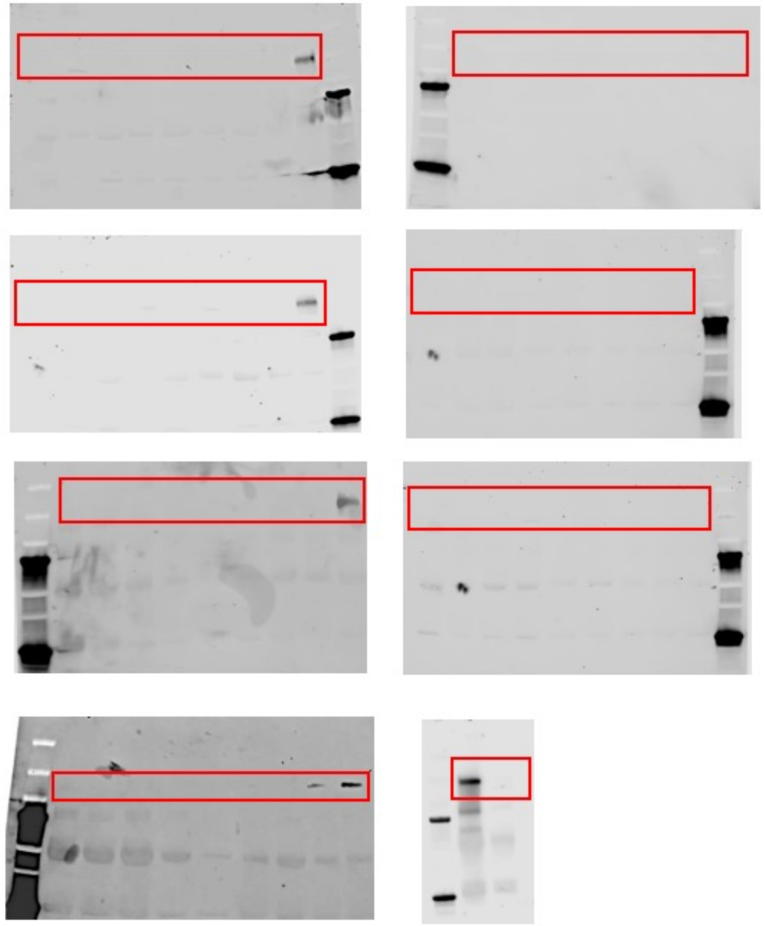

Supplement: Unedited blot and gel images [file jci-136-204727-s152.pdf]
